# Does retirement trigger depressive symptoms? A systematic review and meta-analysis

**DOI:** 10.1017/S2045796021000627

**Published:** 2021-12-01

**Authors:** A. Odone, V. Gianfredi, G. P. Vigezzi, A. Amerio, C. Ardito, A. d'Errico, D. Stuckler, G. Costa

**Affiliations:** 1 Department of Public Health, Experimental and Forensic Medicine, University of Pavia, Pavia, Italy; 2 School of Medicine, University Vita-Salute San Raffaele, Milan, Italy; 3 Department of Neuroscience, Rehabilitation, Ophthalmology, Genetics, Maternal and Child Health, Section of Psychiatry, University of Genoa, Genoa, Italy; 4 Department of Economics and Statistics “Cognetti De Martiis”, University of Turin, Turin, Italy; 5 Department of Epidemiology, ASL TO3, Piedmont Region, Grugliasco, Turin, Italy; 6 Department of Social and Political Sciences, Bocconi University, Milan, Italy; 7 Department of Clinical and Biological Sciences, University of Turin, Turin, Italy

**Keywords:** depression, epidemiology, prevention, retirement, social factors, systematic review and meta-analysis

## Abstract

**Aims:**

Retirement is a major life transition that may improve or worsen mental health, including depression. Existing studies provide contradictory results. We conducted a systematic review with meta-analysis to quantitatively pool available evidence on the association of retirement and depressive symptoms.

**Methods:**

We applied PRISMA guidelines to conduct a systematic review and meta-analysis to retrieve, quantitatively pool and critically evaluate the association between retirement and both incident and prevalent depression and to understand better the potential role of individual and contextual-level determinants. Relevant original studies were identified by searching PubMed, Embase, PsycINFO and the Cochrane Library, through 4 March 2021. Subgroup and sensitivity meta-analyses were conducted by gender, study design (longitudinal *v.* cross-sectional studies), study quality score (QS) and considering studies using validated scales to diagnose depression. Heterogeneity between studies was evaluated with *I*^2^ statistics.

**Results:**

Forty-one original studies met our a priori defined inclusion criteria. Meta-analysis on more than half a million subjects (*n* = 557 111) from 60 datasets suggested a protective effect of retirement on the risk of depression [effect size (ES) = 0.83, 95% confidence interval (CI) = 0.74–0.93], although with high statistical heterogeneity between risk estimates (*χ*^2^ = 895.19, df = 59, *I*^2^ = 93.41%, *p*-value < 0.0001). Funnel plot asymmetry and trim and fill method suggested a minor potential publication bias. Results were consistent, confirm their robustness and suggest stronger protective effects when progressively restricting the included studies based on quality criteria: (i) studies with the highest QS [55 datasets, 407 086 subjects, ES = 0.81, 95% CI = 0.71–0.91], (ii) studies with a high QS and using validated assessment tools to diagnose depression (44 datasets, 239 453 subjects, ES = 0.76, 95% CI = 0.65–0.88) and (iii) studies of high quality, using a validated tool and with a longitudinal design (24 datasets, 162 004 subjects, ES = 0.76, 95% CI = 0.64–0.90). We observed a progressive reduction in funnel plot asymmetry. About gender, no statistically significant difference was found (females ES = 0.79, 95% CI = 0.61–1.02 *v.* men ES = 0.87, 95% CI = 0.68–1.11).

**Conclusions:**

Pooled data suggested that retirement reduces by nearly 20% the risk of depression; such estimates got stronger when limiting the analysis to longitudinal and high-quality studies, even if results are affected by high heterogeneity.

As retirement seems to have an independent and protective effect on mental health and depressive symptoms, greater flexibility in retirement timing should be granted to older workers to reduce their mental burden and avoid the development of severe depression. Retirement may also be identified as a target moment for preventive interventions, particularly primary and secondary prevention, to promote health and wellbeing in older ages, boosting the observed impact.

## Introduction

Globally, the proportion of older adults (>60 years) is estimated to almost double between 2015 and 2050, from about 12% to 22% (United Nations, 2015). As the world population ages, it is critical to promote and support healthy ageing processes to improve societal wellbeing and limit its clinical and economic burden (Dietz *et al*., 1987). The prevalence of late-life depression is 7% among the general older population (Mccall and Kintziger, 2013) and accounts for 5.7% of years lived with disability in those over 60 years old (Killinger, 2012). Depressive symptoms are often overlooked and untreated in older populations, are associated with psychosocial and cognitive decline (Nelson, 2001), and result from a complex interaction between psychological, biological and social factors (Alexopoulos, 2019). One significant determinant that could play a role is transitioning into retirement, whose timing, decision and consequences could be influenced by depressive symptoms, such as loneliness and hopelessness, acting as moderators (Gum *et al*., 2017; Segel-Karpas *et al*., 2018). Retirement is a major life transition that results in social and psychological transformations (Bosse *et al*., 1991), which pose both threats and opportunities for mental health. On the one hand, as a potentially stressful life event, retirement can have adverse repercussions on individual physical and psychological wellbeing (Portnoi, 1981). People lose access to social networks, lifestyles and daily routines, as well as potential stimulation, activity and purposes. Conversely, retirement may reduce work-related exposures and improve physical and mental health through complex mechanisms. These could include an increase in social support and in the time available for leisure and healthy activities, and disconnection from work-related stressors (Van Der Heide *et al*., 2013; Eibich, 2015). These positive health effects were particularly observed among retirees from strenuous jobs (Belloni *et al*., 2016; Blake and Garrouste, 2019; Ardito *et al*., 2020; Carrino *et al*., 2020; Fleischmann *et al*., 2020). Therefore, as we reported in previous research (Vigezzi *et al*., 2021), health behaviours changes (e.g. changes in smoke habit, alcohol consumption, physical activity, time use, social interactions) appeared to be among the most relevant mediators of retirement consequences on olders’ health, affecting life years after the withdrawal from work (Lang *et al*., 2007; Vahtera *et al*., 2009; Celidoni and Rebba, 2017). Nonetheless, current findings are inconclusive. As it has been previously conceptualised (Van Solinge, 2007), health consequences of retiring are influenced by the employment history, the job characteristics (Ardito *et al*., 2020) and the transition to retirement itself, as well as by the availability of socioeconomic resources at the time of retirement and, last but not least, by individuals’ characteristics and appraisal of stress-generating life events (Van Solinge, 2007; Augner, 2018). As a result of such a complex conceptual model, no conclusive evidence exists on the harm-benefit health balance of retirement. In particular, both older and more recent studies have shown contradictory results on the impact of retirement on mental health outcomes (Bossé *et al*., 1987; Salokangas and Joukamaa, 1991; Gall *et al*., 1997; Drentea, 2002; Mein *et al*., 2003; Buxton *et al*., 2005; Gill *et al*., 2006; Mojon-Azzi *et al*., 2007; Van Solinge, 2007; Alavinia and Burdorf, 2008; Vahtera *et al*., 2009; Jokela *et al*., 2010; Westerlund *et al*., 2010).

Here we performed a systematic review and meta-analysis to identify the overall association of retirement with depression. As a second aim, we sought to identify potential modifying individual- and contextual-level factors.

## Methods

We followed the Prepared Items for Systematic Reviews and Meta-Analysis (PRISMA) (Liberati *et al*., 2009; Page *et al*., 2021) and the Meta-analysis Of Observational Studies in Epidemiology (MOOSE) guidelines (Stroup *et al*., 2000).

### Search methods and inclusion criteria

Studies identified searching the electronic databases PubMed/Medline, Embase, PsycINFO and the Cochrane Library through 4 March 2021 were included. The search strategy was first developed in Medline and then adapted for use in the other databases (online Supplementary Table 1). Briefly, we used a combination of free text and exploded MeSH headings, identifying: (i) the concept of ‘retirement/transition to retirement’ and (ii) ‘depression/depressive symptoms’. Further studies were retrieved from manual reference listing of relevant articles and consultation with experts in the field. Details on inclusion and exclusion criteria are reported in Table 1, according to the Population, Exposure, Comparison, Outcomes and Study design (PECOS) framework (Brown *et al*., 2006; Higgins and Green, 2013). Our inclusion criteria were limited to those studies: reporting original data from quantitative analysis, providing effect sizes (ESs) of the association between retirement (exposure of interest) and depression (outcome of interest); natural experiments (Stuckler, 2017; Ronchetti *et al*., 2020), observational studies with prospective, retrospective and cross-sectional designs; and written in English. An extensive definition of retirement and retirement status was used: depending on study design, both retired status and transition to retirement were included as exposure of interest; we considered all retirement types, apart from retirement only for disability, which was excluded. Depression-related outcomes of interest included: depressive symptoms, Diagnostic and Statistical Manual of Mental Disorders (DSM), or International Classification of Diseases scale (ICD)-based diagnosis as major depressive disorder and persistent depressive disorder. We excluded opinion papers (i.e. editorials, narrative reviews, commentaries and letters to the Editor) not providing original data. Systematic reviews were also excluded but screened to retrieve relevant original studies. The review's protocol was drafted and approved by authors before conduction (not archived on public databases).

**Table 1.** *A priori* defined inclusion and exclusion criteria according to the Population (P), Exposure (E), Comparison (C), Outcomes (O) and Study design (S) (PECOS) framework

Search strategy	Details
Inclusion criteria	P: general adult population (male and female)
	E: retirement
	C: still employed
	O: depressive symptoms
	S: original data (all study designs)
Exclusion criteria	E: disability retirement
	O: other mental health outcomes (including anxiety symptoms, post-traumatic stress symptoms)
	S: no original data (opinion papers, review articles, commentaries, letters, protocols, studies without quantitative data)
Language filter	English
Time filter	From inception through 4 March 2021
Database	PubMed/Medline; EMBASE, PsycINFO, Cochrane

### Study selection, data extraction and quality appraisal

Identified studies were independently reviewed for eligibility by two authors (V.G. and G.P.V.) in a two-step process; a first screening was performed based on title and abstract. Then, full texts were retrieved for a second screening. At both stages, disagreements among reviewers were resolved by consensus and by consulting a third senior author (A.O.) when disagreement persisted. Data were independently extracted by two authors (V.G. and G.P.V.), supervised by a third author (A.O.), using an *ad-hoc* developed data extraction spreadsheet. The data extraction spreadsheet was piloted on ten randomly selected papers and modified accordingly. Data extraction included: full reference details, country of study conduction, study design, study setting, study population details, sample size, exposure details, outcomes of interest, including validated assessment tools for depression, and quantitative results, including ESs and corresponding confidence intervals (CIs). Corresponding authors were contacted by e-mail in case of incomplete data. Quality appraisal of included studies was carried out applying the 14-item scoring system developed by Shim *et al*. for population-based studies on retirement as a risk factor (Shim *et al*., 2013). As determined by consensus following the review methodology literature, we consider of high quality the studies with at least ⩾75% of the highest score.

### Data pooling and meta-analysis

We performed descriptive analysis to report and pool the characteristics of included studies using ranges and average values. With regard to the pre-specified outcomes of interest, we would expect variability between studies, e.g. by study design and population. We, therefore, applied random-effects meta-analyses to acquire estimates of the association between retiring and risk of depression/depressive symptoms, rather than to assume a single true value in a fixed-effects approach (Higgins and Green, 2013). Moreover, a random effect model is highly recommended when high heterogeneity is expected or detected. Pooled ESs were calculated as odd ratios (ORs) (Ter Hoeve *et al*., 2020). When the included studies reported ESs as regression beta coefficients with corresponding standard errors (s.e.s), we mathematically converted them into ORs with corresponding CIs (Bland and Altman, 2000; Hailpern and Visintainer, 2003). We also included studies that reported ESs as *χ*^2^ or *ρ* correlation coefficients with corresponding total sample sizes or as mean differences with sample sizes and corresponding correlations. Heterogeneity was assessed using the *I*^2^ statistic (see online Supplementary Table 3 for details) and visual inspection of funnel plots. We performed sensitivity analyses progressively limiting meta-analysis to: (i) high-quality studies; (ii) high-quality studies using validated scales to diagnose depression; (iii) high-quality longitudinal studies using validated scales to diagnose depression. Moreover, we conducted a subgroup meta-analysis by gender strata and study design.

We assessed publication bias with funnel plot visual inspection (Higgins *et al*., 2011) and the Begg and Mazumdar (1994) and Egger *et al*. (1997) tests. A ‘trim and fill’ method was used if publication bias was detected (Duval and Tweedie, 2000; Gianfredi *et al*., 2020) to estimate potential missing studies which contribute to the funnel plot's asymmetry (Sutton *et al*., 2000). This method assumes that the most extreme ES studies have not been reported, biasing the overall ES estimates (Shi and Lin, 2019). Meta-analyses were conducted using ProMeta3^®^ (Internovi, Milan, Italy) software.

## Results

### Characteristics of included studies

**Fig. 1.**
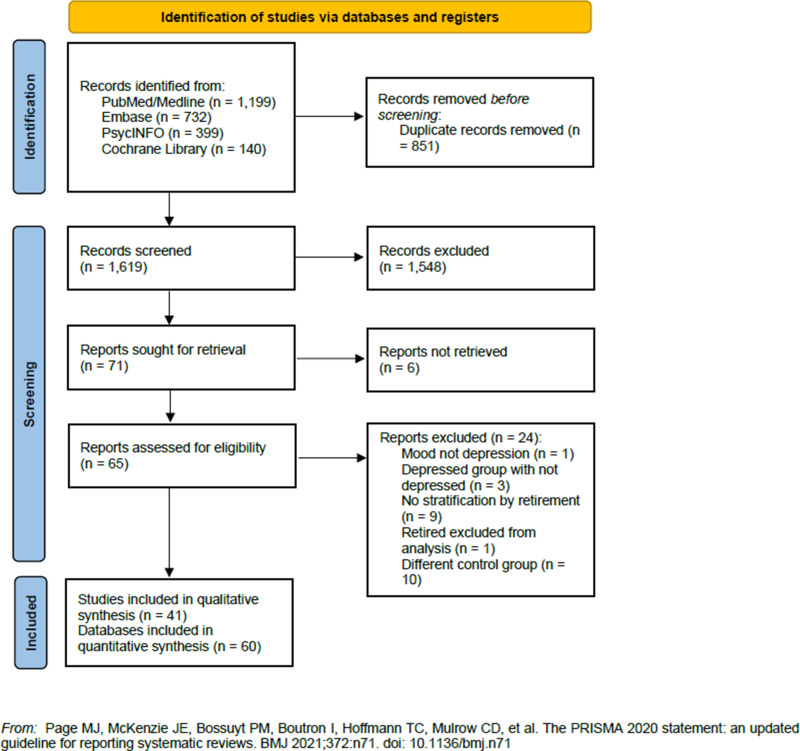
Flow diagram of the studies selection process.

We identified 2470 studies by searching the selected databases and listing references of relevant articles. After removing duplicates, 1619 records were retrieved. Papers were screened and selected, as illustrated in Fig. 1 (1548 records were excluded after first screening; six reports were not retrieved in full text; 24 were excluded with reasons), resulting in 41 papers meeting our inclusion criteria (Farakhan *et al*., 1984; Borson *et al*., 1986; Herzog *et al*., 1991; Pahkala *et al*., 1992; Midanik *et al*., 1995; Reitzes *et al*., 1996; Fernandez *et al*., 1998; Kim and Moen, 2002; Buxton *et al*., 2005; Tuohy *et al*., 2005; Butterworth *et al*., 2006; Mojon-Azzi *et al*., 2007; Alavinia and Burdorf, 2008; Schwingel *et al*., 2009; Coursolle *et al*., 2010; Behncke, 2012; Calvo *et al*., 2013; Choi *et al*., 2013; Gayman *et al*., 2013; Leinonen *et al*., 2013; Airagnes *et al*., 2015, 2016; Bretanha *et al*., 2015; Olesen *et al*., 2015; Belloni *et al*., 2016; Calvó-Perxas *et al*., 2016; Mosca and Barrett, 2016; Park and Kang, 2016; Rhee *et al*., 2016; Heller-Sahlgren, 2017; Shiba *et al*., 2017; Arias-De La Torre *et al*., 2018; Augner, 2018; Fernández-Niño *et al*., 2018; Sheppard and Wallace, 2018; Van Den Bogaard and Henkens, 2018; Anxo *et al*., 2019; Kolodziej and García-Gómez, 2019; Noh *et al*., 2019; Matta *et al*., 2020; Han, 2021). Characteristics of included studies are reported in Table 2. Studies were published between 1984 and 2021, with almost one third (*n* = 12, 29.3%) published in the last 5 years. The majority of the studies (*n* = 21, 51.2%) were conducted in Europe (United Kingdom, *n* = 3; France, *n* = 3; Finland, *n* = 2; Sweden, *n* = 1; Spain, *n* = 1; Switzerland, *n* = 1; Denmark, *n* = 1; Ireland, *n* = 1; Scotland, *n* = 1; multi-centric European studies, *n* = 5) and in the USA (*n* = 13, 31.7%). Four studies were conducted in Asia, one in Brazil, one in Australia; four were multi-centre studies conducted at the global and European level.

**Table 2.** Descriptive characteristics of the included studies stratified by study design and listed in alphabetical order and by study design

Author Year (reference)	Country of study implementation	Study design	Study period	Study population	Sample size (n.) Gender Age	Attrition	Type of retirement	Diagnosis of depression	Validated tool for depression	Effect size (CI95%)	Adjustment	QS
*Cross-sectional study design*												
Alavinia and Burdorf (2008)	Austria, Greece, Denmark, France, Germany, Italy, the Netherlands, Spain, Sweden, Switzerland	Cross-sectional	2004	SHARE study	11 462, F M, 50–64 y	61.8%	Statutory and early retirement	EURO-D	Yes	Moderately depressed: OR = 1.28 (1.08–1.52), *p* < 0.05	Self-perceived health, sex, age, education, BMI, marital status, smoking, drinking, physical activity	15
										Heavily depressed: OR = 2.60 (1.37–4.94), *p* < 0.05		
Anxo *et al*. (2019)	Sweden	Cross-sectional	2014–2015	Combination of Swedish register data and LISA study	3901, F (45%) M (55%), 66–76 y	3878	Prolonged *v.* statutory retirement	Depression index	No	*β* = −0.029 (s.e. = 0.034)	Age, sex, migration status, education, marital status, life expectancy, previous labour market experience, income, previous health	12
Arias-de la Torre *et al*. (2018)	Spain	Cross-sectional	2014–2015	European Health Interview Survey	21 546 F (53.2%) M (46.8%), ⩾18 y	1296	n.a.	PHQ-8	Yes	F: OR = 1.91 (1.31–2.82)	Gender, age, marital status, country of birth, educational level, residence area, employment status, occupational social class	14
										M: OR = 1.68 (0.93–3.03)		
Augner (2018)	Austria, Germany, Sweden, Spain, Italy, France, Denmark, Greece, Switzerland, Belgium, Israel, Czech Republic, Poland, Portugal, Slovenia Estonia, Croatia, Luxembourg	Cross-sectional	2014–2015	SHARE	13 447, F (54.2%) M (45.8%), 60–69 y	n.a.	Statutory and early retirement	EURO-D	Yes	OR = 0.79 (0.71–0.88)^a^	Sex, country	12
Behncke (2012)	UK	Cross-sectional	n.a.	ELSA	Employed: 1247, F (50%) M (50%), mean age 55 y	839	n.a.	CES-D	Yes	*β*_ATET_ = 0.047 (s.e. = 1.22)	Socioeconomic status, sex, age, education, job characteristics, health indicators (subjective and objective health measures, proxy for genetic predisposition, underlying health state)	15
					Retired: 192, F (54%) M (46%), mean age 60.20 y							
Belloni *et al*. (2016)	Austria, Belgium, Denmark, France, Germany, Italy, the Netherlands, Spain, Sweden, Switzerland	Cross-sectional	2004–2013	SHARE	21 960, F (48%) M (52%), 55–70 y	n.a.	Statutory and early retirement	EURO-D	Yes	F: *β*_FE_ = −0.240 (s.e. = 0.082), *p* < 0.01; blue collar F (*n* = 1691) *β*_FE_ = −0.0530 (s.e. = 0.784); white collar F (*n* = 8064) *β*_FE_ = 0.158 (s.e. = 0.285)	Age, marital status, number of grandchildren, household income, health index, regional unemployment rate, waves	14
										M: *β*_FE_ = −0.203 (_S.E._ = 0.062), *p* < 0.01; blue collar M (n = 3399) *β*_FE_ = −0.0534 (_S.E._ = 0.413); white collar M (n = 7212) *β*_FE_ = 0.0892 (_S.E._ = 0.373)		
Borson *et al*. (1986)	USA	Cross-sectional	December 1978	Subjects admitted at The MCCU of the Seattle Veterans Administration Medical Center	404, F (3.5%) M (96.5%), >60 y	513	Disability pension excluded	ZSDS	Yes	Depressed among retired *v.* non-retired: *χ*^2^ = 1.6, *p* = 0.197	Age, sex, marital status, years of education, household income, type of residence, household composition	14
Bretanha *et al*. (2015)	Brazil	Cross-sectional	2008	Subjects living in the 20 basic health units of the urban zone of Bagé, Rio Grande do Sul	1514, F (62.8%) M (37.2%), >60 y	199	n.a.	GDS-15	Yes	OR = 0.77 (0.61–0.97), *p* = 0.026	Sex, age, skin colour, schooling, marital status, economic status	12
Butterworth *et al*. (2006)	Australia	Cross-sectional	1997	National Survey MHWB	4189, F (54%) M (46%), 45–74 y	6452	‘Not in the labour force’ as retirement	CIDI version 2.1, meeting the ICD-10	Yes	F 45–54 retired: OR = 1.20 (0.80–1.80); F 55–64 retired: OR = 0.46 (0.29–0.73); F 65–74 retired: OR = 0.27 (0.17–0.44)	Age group, labour force status, interaction between age and labour force status, main source of income, home ownership, physical health, living situation, partnered, socio-economic status	16
										M 45–54 retired: OR = 2.86 (1.37–6.00); M 55–64 retired: OR = 0.67 (0.35–1.29); M 65–74 retired: OR = 0.42 (0.20–0.85)		
Buxton *et al*. (2005)	UK	Cross-sectional	2000	2000 Psychiatric Morbidity Survey of Great Britain	1875, F (51%) M (49%), 50–64 y	n.a.	Early retirement	CIS-R	Yes	F: OR = 2.6 (0.9–7.4);	Age, tenure, mental health, physical health	15
										M: OR = 4.3 (1.7–11.0)		
Choi *et al*. (2013)	Denmark, Sweden, Austria, France, Germany, Switzerland, Belgium, the Netherland, Spain, Italy, Greece, Israel, Czech Republic, Poland and Ireland	Cross-sectional	2004–2007	SHARE	7238, F (48.3%) M (51.7%) ⩾60 y	8857	Inclusive definition: unemployed, long term sick, disabled, inactive, statutory retirement *v.* paid work	EURO-D	Yes	OR = 1.41 (0.79–2.44)^a^, *p* = 0.24	Age, sex, marital status, education, economic status, country	14
Farakhan *et al*. (1984)	USA	Cross-sectional	n.a.	Elderly black persons	30, F (76%), M (24%) 52–97 y	n.a.	n.a.	DACL	Yes	Data not extractable: reduced risk of depression	n.a.	6
Fernández-Niño *et al*. (2018)	China, Ghana, India, Mexico, Russian Federation and South Africa	Cross-sectional	2007–2009	SAGE study	18 148, F (54%) M (46%), 64–75 y	3262	Retirement with a pension (retirement without a pension and not working due to disability excluded)	World Mental Health Survey version of the Composite International Diagnostic Interview based on ICD-10 (MDE)	Yes	China: F OR = 1.60 (0.73–3.53), *p* = 0.24; M OR = 0.23 (0.08–0.70), *p* = 0.01	Sex, age, marital status, education, belonging to a religious minority, self-report of having some type of health care service, physical disability, multimorbidity, accidents/injuries, living alone, participation in social religious activities, participation in non-religious social activities, horizontal trust, household wealth and country effect	15
					China F 3854 M 3671							
					Ghana F 1469 M 1374					Ghana: F OR = 3.26 (0.84–12.6), *p* = 0.09; M OR = 0.25 (0.07–0.95), *p* = 0.04		
					India F 1870 M 2101					India: F OR = 0.05 (0.01–0.51), *p* = 0.01: M OR = 0.49 (0.16–1.52), *p* = 0.22,		
					Mexico F 1145 M 736					Mexico: F OR = 0.39 (0.04–3.50), *p* = 0.40; M not estimable		
					Russia F 1923 M 1122					Russia: F OR = 0.89 (0.39–2.01), *p* = 0.77; M OR = 1.43 (0.27–7.54), *p* = 0.67		
					South Africa F 1264 M 881					South Africa: F OR = 0.19 (0.04–0.97), p = 0.05; M OR = 0.89 (0.09–8.82), *p* = 0.03		
Heller-Sahlgren (2017)	Austria, Belgium, Denmark, France, Germany, Italy, the Netherlands, Spain, Sweden, Switzerland	Cross-sectional	2004–2012	SHARE	4704, F M, ⩾50 y	3862	Retired but excluding all non-workers in the non-retired category	EURO-D	Yes	EURO-D: *β*_FEIV_ = 1.69 (s.e. = 0.67), *p* < 0.05	Sex, education, occupational physical burden, occupational psychosocial burden	15
										Clinical depression: *β*_FEIV_ = 0.37 (_S.E._ = 0.15), *p* < 0.05		
Herzog *et al*. (1991)	USA	Cross-sectional	May-October 1986	ACL Survey	1332, F M, 55–64 y 510, >65 y 822	2285	Retirement for non-health reasons	CES-D	Yes	55–64y: *β*_OLS_ = −0.14	Gender, race, educational attainment, marital status, current or former occupation, age	15
										>65y: *β*_OLS_ = 0.12 n.s.		
Kolodziej and García-Gómez (2019)	Austria, Belgium, Denmark, France, Germany, Italy, Greece, the Netherlands, Spain, Sweden, Switzerland	Cross-sectional	2004–2013	SHARE	37 333, F (46.3%) M (53.6%), 55–69 y	n.a.	Self-reported retirement status or self-reported permanent absence from the labour force or not having performed any paid work in the past month	EURO-D	Yes	*β*_OLS_ = 0.456 (s.e. = 0.020), *p* < 0.01	Gender, age at the time of the interview, age squared, number of children, education, marital status, area of residence, seasonal dummies, country dummies	15
										F: *β*_IV_ = −1.314 (_S.E._ = 0.341), *p* < 0.01; M: *β*_IV_ = −0.894 (_S.E._ = 0.439), *p* < 0.05		
										White collar: *β*_IV_ = −0.671 (_S.E._ = 0.371), *p* < 0.1; blue collar: *β*_IV_ = −1.792 (_S.E._ = 0.459), *p* < 0.01		
Midanik *et al*. (1995)	USA	Cross-sectional	1985–1987	Northern California Kaiser Permanente Medical Care Program	595, F (43%) M (57%), 55–75 y	400	n.a.	CES-D	Yes	F: RR = 0.5 (0.2–1.2)	Age, gender, marital status, education, baseline mental health	14
										M: RR = 1.0 (0.4–2.4)		
										Total: RR = 0.7 (0.4–1.3)		
Mojon-Azzi *et al*. (2007)	Switzerland	Cross-sectional	1999–2003	Swiss Household Panel	557, F (45%) M (55%), 55–75 y	811	Retired due to old age and retired for other reasons, such as disability or severe illness	Frequency of negative feelings such as depression or anxiety (from 0 = never to 10 = always)	No	OR = 1.1 (0.9–1.3)	Sex, general health at baseline, highest level of education achieved, occupation class, years from official retirement	15
Pahkala *et al*. (1992)	Finland	Cross-sectional	1984	Subjects born in 1923 or earlier and living in a semi-industrialised community in the western part of central Finland	594, F M, ⩾60 y	771	n.a.	ZSDS	Yes	Data not extractable: increased proportion of dysthymic men had retired	Education, occupation, marital status, living conditions, living partners, hobbies, visiting contacts, social participation, appreciation, intimacy of relationships	12
Sheppard and Wallace (2018)	USA	Cross-sectional	2016	Women who had retired part-time or full time from working outside of the home or a home-based business	80, F, 55–81 y	n.a.	Forced or voluntary, part- or full-time retirement for < or = 10 years.	Questions regarding depression	No	*ρ* = −0.116 (for the length of retirement)	n.a.	11
Tuohy *et al*. (2005)	Scotland	Cross-sectional	n.a.	Retired police officers	1334, M, mean age 61 y, range 34–94 y	2669	Early and statutory retirement	HADS	Yes	*ρ* = −0.09 (for the retirement age), *p* < 0.001 *β* = −0.031 n.s.	Anxiety scores, present age, retirement age, retirement type, postretirement work	14
*Prospective cohort studies*												
Airagnes *et al*. (2015)	France	Longitudinal 15 y	1993–2008	GAZEL study	9755, F (20.6%) M (79.4%), mean age 55.4 ± 2.4 y	10 733	n.a.	CES-D	Yes	*β* = −0.378, *p* < 0.001	Age, gender, occupational grade, history of sickness absences for depression, alcohol consumption, Type A personality (competitiveness, sense of urgency, and irritability)	18
Airagnes *et al*. (2016)	France	Longitudinal 25 y	1989–2004	GAZEL study	9242, F (23.7%) M (76.3%), mean age: F 57.0 ± 3.9 y; M 59.4 ± 2.7 y	11 383	n.a.	CES-D	Yes	F: *β* = −0.480, *p* < 0.001	Age, marital status, occupational status, alcohol consumption, self-rated health, CES-D score before retirement, adverse childhood life events	18
										M: *β* = −0.183, *p* = 0.005		
Calvo *et al*. (2013)	USA	Longitudinal 18 y	1992–2010	HRS survey	6624, F (47.3%) M (56.7%), mean age 64.01 ± 6.48 y	3129	Statutory and early retirement	Reduced CES-D	Yes	For emotional health: short-term model: *β*_FEIV_ = −1.54 (s.e. = 0.20), *p* < 0.001; long-term model: *β*_FEIV_ = −1.26 (s.e. = 0.15), *p* < 0.001	Changes to Social Security's full retirement age and unexpected early retirement window offers, age, interaction between retirement and age, wealth, income, marital status, gender, race, education, blue/white collar	18
Calvó-Perxas *et al*. (2016)	Austria, Germany, Sweden, the Netherlands, Spain, Italy, France, Denmark, Switzerland, Belgium, Czech Republic, Slovenia, Estonia	Longitudinal 2 y	2011–2013	SHARE	22 280, F (58.2%) M (41.8%), mean age 64.2 y	15 932	n.a.	EURO-D	Yes	Incident depression after 2 y of retirement: F OR = 1.02 (0.83–1.27); M OR = 1.03 (0.73–1.43)	Age, marital status, employment, education level, number of comorbidities, BMI, BADL, anxiety	16
										Persistent depression after 2 y of retirement: F OR = 1.08 (0.84–1.39); M OR = 0.82 (0.54–1.24)		
Coursolle *et al*. (2010)	USA	Longitudinal, 11 y	1993–2004	Wisconsin Longitudinal Study	2666, F (45%), M (55%), 64–65 y in 2004	7651	Self-reported full and partial retirement	CES-D	Yes	For full retirement: total: *β*_FE_ = −0.97 (s.e. = 0.31), *p* < 0.01; F *β*_FE_ = −0.16 (s.e. = 0.51), n.s.; M *β*_FE_ = −1.53 (s.e. = 0.39), *p* < 0.001	Gender, wages, assets, physical health, educational attainment, family characteristics and relationships, employment characteristics, work-family conflict	19
										For partial retirement: total: *β*_FE_ = −0.61 (s.e. = 0.35), n.s.; F *β*_FE_ = −0.39 (s.e. = 0.60), n.s.; M *β*_FE_ = −0.86 (s.e. = 0.43), *p* < 0.05		
Fernandez *et al*. (1998)	USA	Longitudinal 2 y	1992–1994	Mature men and women residing in a North Carolina metropolitan area	749, F M, 58–64 y	582	Retirement from full-time employment (working less than 35 h a week included)	CES-D	Yes	White men *β* = −0.82, *p* < 0.05	n.a.	15
										African American men *β* = 2.70, *p* < 0.05		
Gayman *et al*. (2013)	USA	Longitudinal 14 y	1994–2008	HRS survey	3264, F M 51–61 y in 1992	9390	Self-reported retirement, excluding disability	CES-D	Yes	Depressed between retired: Whites *χ*^2^ diff. = 5.49, *p* = 0.02	Race, gender, education, age, disability exit, death in the study period	18
					Whites = 2765							
					Blacks = 499					Blacks *χ*^2^ diff. = 0.01, *p* = 0.92		
Han (2021)	USA	Longitudinal 18 y	1998–2016	RAND HRS	9347, F 49.74% M 50.26%, >51 y	n.a.	Retirement as not working or self-identified as completely retired	CES-D	Yes	Retired for non-health reasons: *β*_REWB_ = −0.382 (s.e. = 0.119), *p* < 0.01	Self-rated health, labour force status, transition, age, gender, race, education, occupation type, marital status, household income, household wealth, health insurance coverage, household size	17
										Retired due to poor health: *β*_REWB_ = 1.790 (_S.E._ = 0.117)		
										Continued retirement: *β*_REW__B_ = 0.385 (_S.E._ = 0.053), *p* < 0.001		
Kim and Moen (2002)	USA	Longitudinal 5 y	1994–1999	Cornell Retirement and Well-Being Study	458, F (38%) M (62%), 50–72 y	304	Long-term retirees *v.* newly retired *v.* not-yet retired individuals	CES-D	Yes	F: long-term retired (*n* = 91) *β*_OLS_ = 0.19 (s.e. = 2.94); newly retired (*n* = 33) *β*_OLS_ = −2.09 (s.e. = 3.14)	Income adequacy, subjective health, marital quality, marital conflict, personal control, age, psychological well-being, interaction between psychological well-being and retirement transition, spouse's employment status, interaction between retirement status and spouse's employment status	15
										M: long-term retired (n = 181) *β*_OLS_ = −1.55 (_S.E._ = 1.55); newly retired (n = 47) *β*_OLS_ = −1.67 (_S.E._ = 1.92)		
Leinonen *et al*. (2013)	Finland	Longitudinal 11 y	1997–2007	data from administrative register data from various sources linked together by Statistics Finland	19 877, F M, 57–68 y	n.a.	Disability and old-age retirement	ICD-10	Yes	Mean DDD/3-months period 0.01 (−0.01–0.03); antidepressant drugs use instead of depression	Calendar year, age at retirement, gender, occupational social class, living arrangements	18
Matta *et al*. (2020)	France	Longitudinal 21 y	1989–2014	GAZEL study	17 655, F M, 55.2 y mean age at retirement	1839	Official retirement (retirement due to illness excluded)	CES-D	Yes	*β*_ME_ = −1.704 (s.e. = 0.13), *p* < 0.05	Gender, marital status, occupational status, alcohol occupation, smoking status, time, retirement, interaction between time and retirement, BMI, interactions between BMI and retirement, time and double interaction with retirement and time	18
Mosca and Barrett (2016)	Ireland	Longitudinal 4 y	2009–2013	TILDA	2373, F M, >50 y	4537	Voluntary and involuntary full retirement	CES-D	Yes	Retired due to own ill health: *β*_OLS_ = 2.584 (s.e. = 1.85), *p* < 0.10	Death of a close relative or a friend, stop participating in a group, changes in functional capacity, chronic illness, self-reported health and vision, changes in income	16
										Retired involuntary: *β*_OLS_ = 2.212 (_S.E._ = 1.71), *p* < 0.10		
										Retired voluntarily: *β*_OLS_ = 0.674 (_S.E._ = 1.56), n.s.		
Noh *et al*. (2019)	Korea	Longitudinal 2 y	2010–2012	Korea Longitudinal Study of Aging	7134, F M, ⩾45 y	8272	Retired *v*. currently working or currently not working but looking for a job	CES-D10	Yes	Total: *β*_OLS_ = 0.12 (s.e. = 0.19), 95% CI −0.26–0.50	Age, gender, education, marital status, self-rated health status, urbanity, CES-D10 score in 2010	17
										F: *β*_OLS_ = −0.56 (_S.E._ = 0.29), 95% CI −1.12–0.01		
										M: *β*_OLS_ = 0.90 (_S.E._ = 0.26), *p* < 0.001, 95% CI 0.40–1.41		
Olesen *et al*. (2015)	Denmark	Longitudinal 6 y	2000–2006	Danish national registers/administrative data 2000–2006	245 082, F (49%) M (51%), n.a.	7134	Statutory old-age retirement (disability pension excluded)	Hospital treatment for depression (ICD-10)	No	Total: OR = 1.15 (95% CI 0.98–1.35)	Sex, cohabitation, disposable income, level of education, area of residence	19
										F: OR = 1.23 (95% CI 0.98–1.54)		
										M: OR = 1.07 (95% CI 0.86–1.34)		
Park and Kang (2016)	Korea	Longitudinal 6 y	2006–2012	Korea Longitudinal Study of Aging	5937, F (50%) M (50%), mean age 59.60 ± 10.02 y	4317	Voluntary and involuntary retirement	CES-D10	Yes	F: voluntary retirement HR = 1.361 (1.051–1.762); involuntary retirement HR = 1.584 (1.216–2.062)	Age, property, household income, perceived health status, medical disability	19
										M: voluntary retirement HR = 1.255 (0.987–1.596); involuntary retirement HR = 1.310 (1.063–1.613)		
Reitzes *et al*. (1996)	USA	Longitudinal 2 y	1992–1994	Carolina Health and Transitions Study	757, F (52%) M (48%), range 58–64 y	69	n.a.	CES-D	Yes	*β* = −0.132 (s.e. = −1.655), *p* < 0.001	Poor health, age, race, marital status, gender, income, education, occupation, worker commitment, worker identity, depression in 1992	16
Rhee *et al*. (2016)	USA	Longitudinal 4 y	2006–2010	RAND HRS survey with Participant Lifestyle Questionnaire	1195, F (48%) M (52%), >50 y, mean age 61.89 y	828	Multicategorical model: transition to voluntary and involuntary retirement	CES-D	Yes	Transition to involuntary retirement: *β* = 0.09 (s.e. = 0.07)	Control over financial situation, positive family relationships, negative family relationships, social integration	18
										Transition to voluntary retirement: *β* = −0.04 (_S.E._ = 0.04)		
Schwingel *et al*. (2009)	Singapore	Cross-sectional	2004–2007	Singapore Longitudinal Ageing Studies	2716, F M, ⩾55 y; retired = 1360, workers = 201	92	Retired and non-volunteering *v*. still working and retired and volunteering *v*. still working	GDS-15	Yes	Retired and not volunteering: mean = 3.17 (s.e. = 0.11), *p* = 0.012; retired and volunteering: mean = 2.68 (s.e. = 0.18), p = 0.71; still working: mean = 2.76 (s.e. = 0.18)	Age (<62 or ⩾62), education, gender, social network and support, general health status, physical functioning	19
		Longitudinal 2 y			1754, F M, ⩾55 y	1054				Retired and not volunteering: baseline mean = 1.91 ± 2.72, follow-up mean = 1.16 ± 2.09, p = 0.03; retired and volunteering: baseline mean = 1.27 ± 2.07, follow-up mean = 0.65 ± 1.37; still working: baseline mean = 1.39 ± 2.04, follow-up mean = 0.58 ± 0.99, p = 0.58	Age (<62 or ⩾62), education, gender, social network and support, general health status, physical functioning, interval between baseline and follow-up	
Shiba *et al*. (2017)	Japan	Longitudinal 3 y	2010–2013	Japan Gerontological Evaluation Study	62 437, F (46%) M (54%), ⩾65 y, mean age 72.9 y	n.a.	Still at work *v*. retired *v*. long-term retired	GDS-15	Yes	F: retired *β* = 0.28 (95% CI 0.12–0.44); long-term retired *β* = 0.05 (95% CI −0.03–0.14)	Changes in social contacts and social support, occupational class, social participation, household income, marital status, instrumental activities of daily living, incidence of serious illnesses, family caregiving	17
										M: retired *β* = 0.33 (95% CI 0.21–0.45);long-term retired *β* = 0.10 (95% CI 0.02–0.18)		
van den Bogaard and Henkens (2018)	20 European countries and Israel	Longitudinal 2 y	2011–2013	SHARE	9092, F M, range 50–70 y	6040	Voluntary retirement	EURO-D	Yes	*β*_OLS_ = −0.10 (s.e. = 0.03), *p* < 0.01	Physical and psychological job demand, gender, partner T1, educational level, work hours T1, household income T1, age, country, physical and mental health score T1, interaction between health situation T1 and retirement	19

*ρ*, Pearson correlation; ACL, Americans' Changing Lives; ATET, Average Treatment Effect on the Treated; BALD, Basic Activities of Daily Living; BMI, Body Mass Index; BDI, Beck Depression Inventor; CES-D, Center for Epidemiologic Studies Depression Scale; CIS-R, Clinical Interview Schedule-Revised; CCRC, Continuing Care Retirement Community; CI, Confidence Interval; CIDI, Composite International Diagnostic Interview version 2.1; DACL, Depression Adjective Check List; DDD, Defined Daily Dose; ELSA, English Longitudinal Study of Ageing; EURO-D, Euro Depression-scale; F, Female; FE, Fixed Effects; FEIV, Fixed Effects Instrumental Variables; GAZEL, Gaz et Electricité; GDS, Geriatric Depression Scale; HADS, The Hospital Anxiety and Depression Scale; HR, hazard ratio; HRS, Health and Retirement Study; ICD, International Classification of Diseases scale; IV, Instrumental Variables; LISA, Longitudinal Integration Database for Health Insurance and Labour Market Studies; M, Male; MCCU, Medical Comprehensive Care Unit; MHWB, National Survey of Mental Health and Well-Being; n.a., not available; n.s., not significant; OLS, Ordinary Least Squares; OR, Odd Ratio; PHQ, Patient Health Questionnaire; QS, Quality Score; RR, relative risk; SAGE, Study on Global Ageing and Adult Health; s.e., Standard Error; SHARE, Survey on Health and Ageing in Europe; TILDA, The Irish Longitudinal Study on Ageing; UK, United Kingdom; USA, United States of America; y, years; ZSDS, Zung Self-rating Depression Scale.

a Odds ratios and corresponding confidence intervals were calculated as the reverse odds ratios for the association between depression and employment compared to retired people.

Nineteen (46.3%) studies were longitudinal studies; their follow-up time ranged from 2 to 25 years, with most of them (*n* = 12, 60.0%) having less than 10 years of follow-up. Most of the longitudinal analyses were derived from the Survey on Health and Ageing and Retirement in Europe (SHARE, *n* = 8), followed by the Health and Retirement Study (HRS, *n* = 4) and the Gaz et Electricité cohort study (GAZEL, *n* = 3). Twenty-one studies (51.2%) had a cross-sectional study design, while only one study reported both cross-sectional and longitudinal data (Schwingel *et al*., 2009). Overall, sample sizes of included studies ranged from 30 (Farakhan *et al*., 1984) to 245 082 subjects (Olesen *et al*., 2015) (mean: 14 423 subjects, median: 4189 subjects); longitudinal studies sample sizes ranged between 458 and 245 082 subjects (mean: 21 884 subjects, median: 7134 subjects). The majority of included study populations’ age ranged between 45 and 80 years (*n* = 38, 92.7%). One study included only males (Tuohy *et al*., 2005) and one only females (Sheppard and Wallace, 2018). Details on study populations are reported in Table 2, which also reports information on the type of retirement, available for 76% of studies, and outcomes assessment.

More than ninety per cent of included studies used validated tools to diagnose depression-related outcomes, including the Center for Epidemiologic Studies Depression scale (CES-D) in 17 studies (41.5%), the Euro Depression-scale (EURO-D) in eight studies (19.5%), the Geriatric Depression Scale (GDS) in three studies (7.1%) and the Zung Self-rating Depression Scale (ZSDS) in two studies (4.9%). The International Classification of Diseases-10 (ICD-10) was used to identify depression-related conditions in three studies (7.1%). The Patient Health Questionnaire-8 (PHQ-8), the Hospital Anxiety and Depression Scale (HADS), the Composite International Diagnostic Interview (CIDI), the Depression Adjective Check List (DACL) and the Clinical Interview Schedule-Revised (CIS-R) were used in only one study each (Table 2). Three studies (7.1%) used non validated tools to identify depression-related outcomes (Mojon-Azzi *et al*., 2007; Sheppard and Wallace, 2018; Anxo *et al*., 2019). Included studies’ quality score (QS) is also reported in Table 2. The mean QS was 15.5/20. The lowest QS was 6 (Farakhan *et al*., 1984), whereas the highest was 19 (Schwingel *et al*., 2009; Olesen *et al*., 2015; Park and Kang, 2016; Van Den Bogaard and Henkens, 2018). Question 7 [Is retirement a main effect, co-variable, confounder, or interaction in the study?] (*n* = 15) and Question 13 [Was the loss to follow-up appropriately addressed and/or adequately described in the study?] (*n* = 11) reported the lowest scores (for details on quality appraisal, see online Supplementary Table 2).

### Retirement and depression: qualitative reporting

Overall, more than one third (*n* = 15, 36.6%) of included studies reported a statistically significant negative association between retirement and depression (i.e. retirement decreased the risk of depression) (Farakhan *et al*., 1984; Tuohy *et al*., 2005; Butterworth *et al*., 2006; Schwingel *et al*., 2009; Coursolle *et al*., 2010; Calvo *et al*., 2013; Airagnes *et al*., 2015, 2016; Bretanha *et al*., 2015; Belloni *et al*., 2016; Augner, 2018; Van Den Bogaard and Henkens, 2018; Kolodziej and García-Gómez, 2019; Matta *et al*., 2020; Han, 2021), 14.6% (*n* = 6) reported a positive association (Pahkala *et al*., 1992; Alavinia and Burdorf, 2008; Park and Kang, 2016; Heller-Sahlgren, 2017; Shiba *et al*., 2017; Arias-De La Torre *et al*., 2018), while 48.8% (*n* = 20 studies) did not report statistically significant associations between retirement and depression (Borson *et al*., 1986; Herzog *et al*., 1991; Midanik *et al*., 1995; Reitzes *et al*., 1996; Fernandez *et al*., 1998; Kim and Moen, 2002; Buxton *et al*., 2005; Mojon-Azzi *et al*., 2007; Behncke, 2012; Choi *et al*., 2013; Gayman *et al*., 2013; Leinonen *et al*., 2013; Olesen *et al*., 2015; Calvó-Perxas *et al*., 2016; Mosca and Barrett, 2016; Rhee *et al*., 2016; Fernández-Niño *et al*., 2018; Sheppard and Wallace, 2018; Anxo *et al*., 2019; Noh *et al*., 2019). The reported ESs included: *β* coefficients (*β*) (*n* = 19), ORs (*n* = 11), *χ*^2^ (*n* = 3), relative risks (RRs) (*n* = 1), hazard ratios (HRs) (*n* = 1) and mean differences (*n* = 2). Almost all included studies reported adjusted effect estimates (i.e. accounting for age, gender, education, health and marital status; details on multivariate models’ adjustments are reported in Table 2). Two studies reported separate data based on the severity of depression (Alavinia and Burdorf, 2008; Heller-Sahlgren, 2017); two studies reported separate data for short-term (incident) and long-term (persistent) depression (Calvo *et al*., 2013; Calvó-Perxas *et al*., 2016); two studies reported separate data by age group (Herzog *et al*., 1991; Butterworth *et al*., 2006). Some studies differentiated the analysis by type of retirement, distinguishing between full and partial retirement (Coursolle *et al*., 2010), retirement for health and non-health reasons (Han, 2021), long-term and new retirement (Kim and Moen, 2002; Shiba *et al*., 2017), voluntary and involuntary retirement (Mosca and Barrett, 2016; Park and Kang, 2016; Rhee *et al*., 2016), or considered retirement jointly with volunteering (Schwingel *et al*., 2009). One study reported separate data for cross-sectional and longitudinal analysis (Schwingel *et al*., 2009), as reported above.

Fourteen studies reported separate data for men and women (Midanik *et al*., 1995; Kim and Moen, 2002; Buxton *et al*., 2005; Butterworth *et al*., 2006; Coursolle *et al*., 2010; Olesen *et al*., 2015; Airagnes *et al*., 2016; Belloni *et al*., 2016; Calvó-Perxas *et al*., 2016; Park and Kang, 2016; Shiba *et al*., 2017; Arias-De La Torre *et al*., 2018; Fernández-Niño *et al*., 2018; Kolodziej and García-Gómez, 2019); two studies reported separate data based on ethnicity (Gayman *et al*., 2013; Fernandez *et al*., 1998); one study reported independent results based on the country (Fernández-Niño *et al*., 2018), so they were considered separately. Among included studies, data from two studies were not extractable (Farakhan *et al*., 1984; Pahkala *et al*., 1992). Three studies did not report ESs and CIs (Herzog *et al*., 1991; Fernandez *et al*., 1998) or outcomes comparable to other works (Leinonen *et al*., 2013) and were not included in the quantitative analysis.

### Retirement and depression: quantitative reporting and meta-analysis

**Fig. 2.**
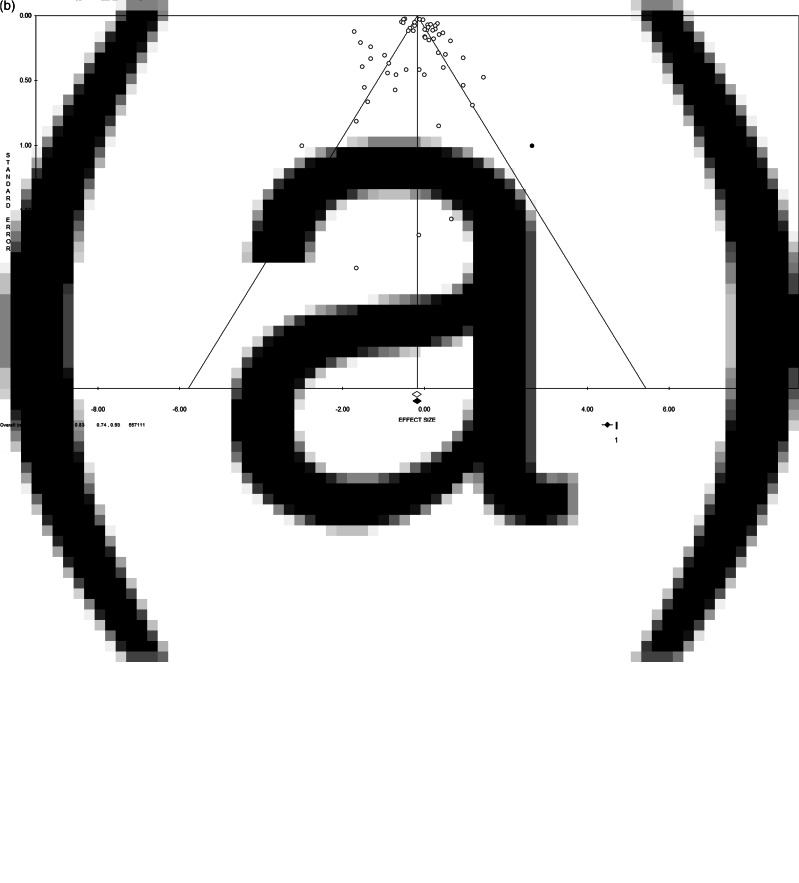
(a) Forest plot and (b) funnel plot (after trim and fill method) of the meta-analysis assessing the association between retirement and depression. ES, effect size; CI, confidence interval.

Quantitative pooling of effect estimates was conducted on a total of 557 111 subjects from 60 different databases. Overall, the pooled ES for the risk of depression when retired is 0.83 (95% CI = 0.74–0.93, *p*-value = 0.001, Fig. 2a), with high statistical heterogeneity (*χ*^2^ = 895.19, df = 59, *I*^2^ = 93.41, *p*-value < 0.001). The funnel plot resulted slightly asymmetrical at visual inspection, showing a low potential for publication bias, not confirmed by Egger's linear regression test (Intercept 0.53, *t* = 0.78, *p*-value = 0.439). Moreover, the ES change after the trim and fill method was minor [0.84 (95% CI = 0.75–0.94)], and two studies were trimmed in the lower right quarter of the funnel plot (Fig. 2b), suggesting few studies of poor quality could be missing.

**Fig. 3.**
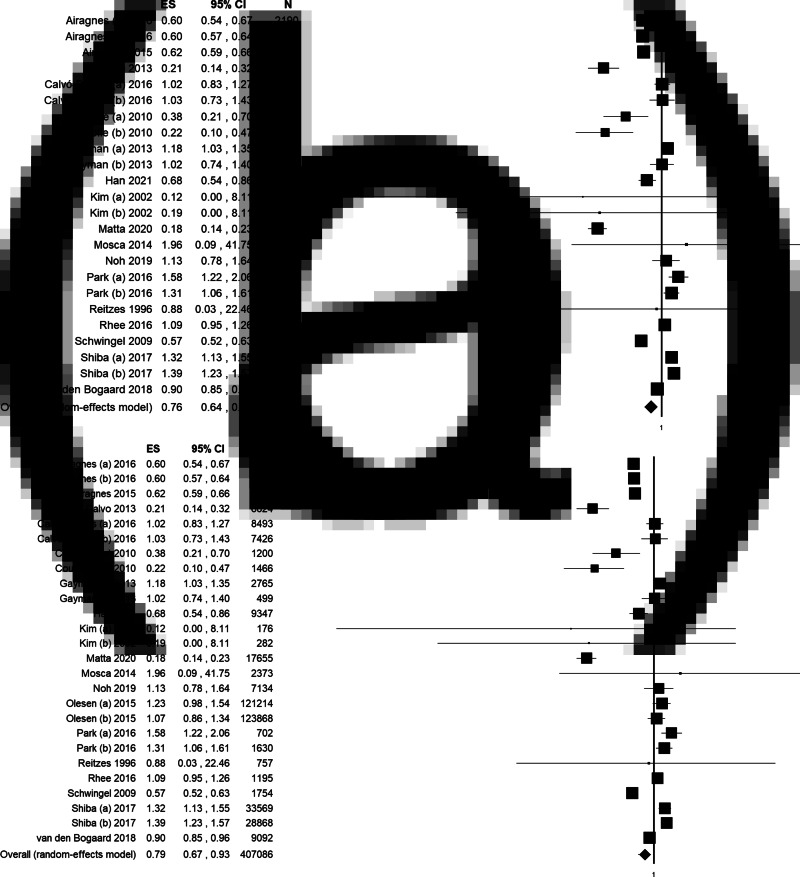
Forest plot of subgroups meta-analysis assessing the association between retirement and depression limited to: (a) studies with a quality score (QS) equal or higher than 15, using validated diagnostic tools and with a longitudinal study design; (b) longitudinal studies. ES, effect size; CI, confidence interval.

Results of the sensitivity and subgroup analyses are summarised in Table 3. We performed a sensitivity analysis, progressively increasing the quality of included studies in order to test our overall results’ consistency. First, we limited the analysis to studies of the highest quality (QS equal or higher than 15): 47 datasets and 485 092 subjects were included in the meta-analysis, reporting a consistent statistically significant association between retirement and decreased risk of depression (ES = 0.79, 95% CI = 0.68–0.91, *p*-value = 0.001, online Supplementary Fig. 1a). Then, we limited the analysis to studies with high QS and using validated assessment tools to diagnose depression. In this analysis, 44 datasets were included, for a total of 239 453 subjects, strengthening the significant association between retirement and decreased risk of depression (ES = 0.76, 95% CI = 0.65–0.88, *p*-value < 0.001, online Supplementary Fig. 1b). Finally, only studies (i) with a QS equal or higher than 15, (ii) using validated assessment tools to diagnose depression and (iii) with a longitudinal study design were included. We report a statistically significant association between retirement and depression (ES = 0.76, 95% CI = 0.64–0.90, *p*-value = 0.001, Fig. 3a) based on 24 datasets and 162 004 subjects. High statistical heterogeneity and slight visual asymmetry of the funnel plot were observed at each step of the analysis (Table 3), with the exception of the last one restricted to longitudinal studies of the highest quality, when estimated ES did not change with the trim and fill method.

**Table 3.** Results of overall, sensitivity and subgroup analyses

Type of analysis	N. of included datasets	ES	95% CI, *p*-value	N. of participants	*χ*^2^; df	*I*^2^	*p*-value	Intercept^a^	*t*-value^a^	*p*-value^a^
Overall	60	0.83	(0.74; 0.93), 0.001	557 111	895.19; 59	93.41	<0.001	0.53	0.78	0.439
*Sensitivity analysis*										
QS ⩾15	47	0.79	(0.68; 0.91), 0.001	485 092	808.42; 46	94.31	<0.0001	0.52	0.65	0.520
QS ⩾15 + validated tool to diagnose depression	44	0.76	(0.65; 0.88), 0.0001	239 453	763.78; 43	94.37	<0.0001	0.33	0.41	0.687
QS ⩾15+ validated tool to diagnose depression + longitudinal design	24	0.76	(0.64; 0.90), 0.001	162 004	652.18; 23	96.47	<0.001	0.85	0.52	0.607
*Subgroup analysis by study design*										
Longitudinal	26	0.79	(0.67; 0.93), 0.004	407 086	681.14; 25	96.33	<0.001	1.18	0.76	0.455
Cross-sectional	33	0.89	(0.76; 1.04), 0.136	139 484	161.43; 32	80.18	<0.001	−0.24	−0.48	0.638
*Subgroup analysis by gender*										
Women	21	0.79	(0.61; 1.02), 0.074	219 655	189.48; 20	89.44	<0.001	−0.25	−0.25	0.805
Men	20	0.87	(0.68; 1.11), 0.258	223 840	252.80; 19	92.48	<0.001	0.99	0.93	0.366

df, degree of freedom; ES, Effect Size; N., number; QS, quality score

a Egger's linear regression test.

These results appeared to be dragged by longitudinal studies as, when considering data from longitudinal studies only (26 datasets, 407 086 subjects), a statistically significant association between retirement and depression was equally found (ES = 0.79, 95% CI = 0.67–0.93, *p*-value = 0.004), with high statistical heterogeneity (*χ*^2^ = 681.14, df = 25, *I*^2^ = 96.33, *p*-value < 0.001), but no publication bias, as confirmed by funnel visual inspection and Egger's test (Intercept 1.18, t = 0.76, *p*-value = 0.455, Table 3, Fig. 3b). On the contrary, no statistically significant association resulted when quantitative pooling was limited to cross-sectional studies (33 datasets, 139 484 subjects) (Table 3, online Supplementary Fig. 2a). In this case, publication bias was suggested by funnel plot visual inspection.

Gender-strata meta-analyses are reported in online Supplementary Fig. 2b and 2c. When only considering women, the analysis included 21 datasets and a total of 219 655 subjects, reporting no statistically significant association between retirement and depression (pooled ES = 0.79, 95% CI = 0.61–1.02, *p*-value = 0.074) and high heterogeneity (Table 3). About men, the analysis included 20 datasets, for a total of 223 840 participants, reporting a pooled ES of 0.87 (95% CI = 0.68–1.11, *p*-value = 0.258) and high heterogeneity between studies (Table 3). In both cases, evidence of publication bias was suggested by funnel plot.

## Discussion

Pooled data from 41 original studies and more than half a million subjects suggested that retirement or transition to retirement reduce by nearly 20% the risk of depression or depressive symptoms; such estimates remain consistent when limiting the analysis to longitudinal and high-quality studies.

Before interpreting our findings further, we must account for the considerable heterogeneity among the included studies, which might limit the generalisability of pooled effect estimates. To overcome this and test the results level of strength, we first applied a random-effect model. Secondly, we conducted sensitivity and stratified meta-analyses by study design and QS. The reasons behind the high level of heterogeneity among the included studies are to be explored in light of, on one side, the wide variety of studies’ designs, settings and populations, definitions and methodological quality and, on the other side, of the complex, multi-determinant and multi-mediator relationship between the process of retirement and mental health and wellbeing (Pesaran *et al*., 1999; Rabe-Hesketh and Skrondal, 2008; Behncke, 2012; Oksanen and Virtanen, 2012; Insler, 2014; Eibich, 2015). We could not retrieve further evidence on the reasons: even excluding one dataset at a time in the meta-analysis to identify potential outliers, heterogeneity persisted (online Supplementary Table 3). However, sensitivity analyses confirmed the results’ consistency.

Despite half of the retrieved studies being cross-sectional, which did not allow us to explore causality, they accounted for less than one-third of included subjects. Another limitation to consider is that duration of retirement was not reported in most studies, so we could not differentiate among the potential risk of depression for short- or long-term exposure to retirement. A subgroup analysis considering the work before retiring was not possible since only two included studies stratified results for this variable (Belloni *et al*., 2016; Kolodziej and García-Gómez, 2019). Lastly, even if most of the analysed data came from administrative databases or surveys designed for other purposes, some studies had small sample sizes with poor precision in effect estimates.

To the best of our knowledge, this is the first systematic review and meta-analysis pooling all original studies investigating the association of retirement with prevalent and incident depression. We used a comprehensive range of databases and search terms to maximise the number of studies retrieved and minimise the chance of publication bias. Besides, further studies were retrieved from the reference listing of relevant articles. Such a comprehensive and rigorous summary of the available evidence offers several meaningful insights, valuable to plan, implement and evaluate public health and preventive strategies, public policies, as well as future avenues of research.

Despite the well-known assumption that considers retirement as a potentially stressful life event (Kremer, 1985; Ekerdt, 1987; Salokangas and Joukamaa, 1991), one of our review's critical findings is that retiring does not necessarily harm an individual's mental health but possibly decrease the risk of depression, as a balance of contextual and individual-level variables impact on such association. In conceptual frameworks proposed in the ageing research literature (Van Solinge, 2007), these variables were categorised into: (i) characteristics of the retirement transition, (ii) characteristics of the job, (iii) access to resources, (iv) individual appraisal and (v) gender.

Characteristics of the transition refer to the type and conditions of retirement, which were available in 76% of included studies. For instance, we report different impacts on depression between voluntary and involuntary retirement, with the more considerable impact of the latter (Mosca and Barrett, 2016), suggesting elements of desirability and degree of control might play a role in the association (Van Solinge, 2007).

There is extensive literature on how employment characteristics influence health after retirement (Hernberg, 2001; Robroek *et al*., 2013; De Wind *et al*., 2014, 2015; Soh *et al*., 2016; Ardito *et al*., 2020). As emerges from original data, among job characteristics, employment history, time pressure, workload and physical demand may impact the risk of mental health disorders’ onset after retirement (Thoits, 1983; Shultz *et al*., 1998).

With reference to resources, access to social and financial resources around retirement might compensate and mitigate the impact of lifestyle changes and the psychological consequences of retiring. We reviewed data where the risk of depression at retirement is differentially distributed by household socioeconomic status (Arias-De La Torre *et al*., 2018), marital and family relations (Park and Kang, 2016), social engagement (Sabbath *et al*., 2015; Shiba *et al*., 2017): as the studies suggest, reliable financial resources, social networks and marriage can mitigate negative health repercussions of retirement (Deeg and Bath, 2003).

Concerning individual appraisal, personality characteristics influence the meaning assigned to retirement and the ability to cope with this change. Negative expectations and fears about retirement are more likely related to adverse repercussions on individuals’ wellbeing (Barnes-Farrell, 2003). Moreover, having confidence in coping with changes determines fewer difficulties in adjusting to retirement (Van Solinge and Henkens, 2005).

Regarding gender, differences in primary role between women and men, at home and work, respectively, could explain differences in adapting to the event and in health outcomes by gender (Moen, 1996), but need to be further explored.

Overall and sensitivity analyses results are consistent with other reviews on the topic. Van Der Heide *et al*. (2013) focused on mental health and antidepressant use in longitudinal studies. They registered an improvement in mental health shortly after retirement, possibly linked to work pressure reduction, even if with gender differences. Schaap *et al*. (2018) analysed the health effects of an exit from work across different socioeconomic groups. They found out that, despite significant heterogeneity, withdrawal from work had more positive effects among employees with a higher socioeconomic status than with a lower position. On the contrary, a systematic review was previously conducted on the effects of working or volunteering beyond statutory retirement ages on mental health by Maimaris *et al*. (2010); they suggested that, through the mechanism of maintaining a productive societal role with a continued income and social support, working beyond retirement age might be beneficial for mental health. Nevertheless, the benefits were not universal, but they varied greatly by lifestyles, self-esteem and socioeconomic status.

### Implications for public health policies and practice

Regarding public health and preventive strategies, we demonstrated that, besides other factors influencing the risk of late-life depression, transition to retirement, as a life event that almost the entire population experience at some point (Clark and Oswald, 1994), has an independent effect in itself. The transition is differentially distributed by contextual and individual-level characteristics and, as such, could be identified as a target point for mental health prevention, including both primary and secondary interventions. We claim that primary prevention interventions, aimed at promoting healthy lifestyles and supporting social roles, could be effectively directed towards subjects who do not benefit from retirement flexibility and its protective effect on short- and long-term risk of late-life depression (Smit *et al*., 2006; Barnett *et al*., 2012; Lindwall *et al*., 2017). As life-course transitions tend to bring along lifestyle changes, synchronising them with public health interventions might be a successful approach (Ben-Shlomo and Kuh, 2002; Werkman *et al*., 2010; Heaven *et al*., 2013, 2016). Along the same lines, secondary prevention, including early depressive symptoms detection, could be effectively targeted to older workers still employed, with particular reference to interventions implemented at the primary care level (Okereke *et al*., 2013; Costantini *et al*., 2021).

About public policies, our data complement the accumulating evidence on the impact of pension reforms on health and mental health (Eibich, 2015; Carrino *et al*., 2020), suggesting that older workers should be granted greater flexibility in the timing of retirement in order to reduce their mental burden and avoid the development of severe depression. As many countries are implementing budget reductions to social welfare (Hall and Soskice, 2001), it is crucial to retrieve solid evidence on how different retirement policies might impact healthy ageing to balance money saved from cuts to pension systems with direct and indirect costs passed onto healthcare and social support systems. Although our review only focuses on mental health, the burden of mental health and, in particular, of depression is known to be associated with the burden of chronic physical conditions that significantly affect people's quality of later life, their demands for healthcare and other publicly funded services, generating significant societal consequences (Bech *et al*., 2011; Hughes *et al*., 2011; Rechel *et al*., 2013).

### Recommendations for future research

Concerning research, it clearly emerges from our analysis that, in order to reduce heterogeneity and accumulate solid evidence, shared methodological standards and definitions should be followed in the future. More extended longitudinal studies should be preferred so as to reduce inverse causality issues and might help disentangle and quantify the different components that mediate the effects of retirement on the risk of depression and its determinants and monitor such association's temporal evolution. It would also be necessary to further differentiate between contextual and individual characteristics to adapt coping strategies at the public health and clinical levels. Special attention should be paid to health inequalities to investigate better socioeconomic status indicators role in the relationship between retirement and health (Adler *et al*., 1994) and address the impact of specific policies focusing on health promotion for disadvantaged groups (Rechel *et al*., 2013). Stratifying results by job and retirement type and by socioeconomic status might be helpful to fill the gaps in current literature.

## Conclusions

As a matter of fact, despite current trends in extending working lives, life expectancy after regular retirement is projected to grow faster than increases in the pension age, reaching 20.3 years for men and 24.6 years for women in 2050 (OECD, 2011). Therefore, from a societal, welfare and public health perspective, it is essential to invest in ‘third age’ health and wellbeing (Crimmins, 2015). In a progressively ageing society, strengthened efforts are needed to make health interests count in welfare and pension policies and promote health protection after retirement (Moen, 1996). We call for a coordinated advocacy action to identify retirement as a gateway for healthy lifestyles and an entry point for mental health prevention. Multidisciplinary collaborations between social sciences, public and community health, preventive medicine and psychiatry could be fruitfully put in place to generate much-needed evidence on the determinants, mediators and effect modifiers of the association between retirement and depression, as well as to design preventive interventions targeting older workers.

## Supplementary material

For supplementary material accompanying this paper visit http://dx.doi.org/ 10.1017/S2045796021000627. click here to view supplementary material

## Data

The datasets supporting the conclusions of this study are available from the corresponding author upon request.
