# Supplementary material for: Does retirement trigger depressive symptoms? A systematic review and meta-analysis
Source: Epidemiol Psychiatr Sci. 2021 Dec 1;30:e77. doi: 10.1017/S2045796021000627 (PMC8679838; doi:10.1017/S2045796021000627)
Supplement: Supplementary file 1 [file S2045796021000627sup001.docx]

**Supplementary Table 1:** Search strategy in PubMed/MEDLINE, adapted for use with other databases.

| **SET** | **PubMed** |
| --- | --- |
| 1  2 | retire* [tiab]  retirement [MeSH Terms] |
| 3 | Sets 1-2 were combined with “OR” |
| 4  5  6  7  8 | depression [MeSH Terms]  depression [Title/Abstract]  depressive [Title/Abstract]  depression [Text Word]  depressive [Text Word] |
| 9 | Sets 4-8 were combined with “OR” |
| 10  11  12  13 | review  case reports  letter  editorial |
| 13 | Sets 10-13 were combined with “OR” |
| 14 | Sets 3 and 9 were combined with “AND” |
| 15 | Sets 14 and 13 were combined “NOT” |
| 16 | Set 15 was filtered by: English language, Humans, To 4^th^ March 2021, >19 years old |

**Supplementary Table 2:** Quality assessment of the included studies, using Quality Assessment Question by Shim et al. (2013).

| **Study** | **Q1** | **Q2** | **Q3** | **Q4** | **Q5** | **Q6** | **Q7** | **Q8** | **Q9** | **Q10** | **Q11** | **Q12** | **Q13** | **Q14** | **Total QS** |
| --- | --- | --- | --- | --- | --- | --- | --- | --- | --- | --- | --- | --- | --- | --- | --- |
| **Airagnes, 2015** | No | 1 | 2 | 2 | 1 | 0 | 1 | 1 | 3 | 1 | 0 | 2 | 1 | 3 | 18 |
| **Airagnes, 2016** | No | 1 | 2 | 2 | 1 | 0 | 1 | 1 | 3 | 1 | 0 | 2 | 1 | 3 | 18 |
| **Alavinia, 2008** | No | 1 | 2 | 2 | 0 | 2 | 0 | 1 | 0 | 1 | 1 | 2 | 0 | 3 | 15 |
| **Anxo, 2019** | No | 1 | 2 | 1 | 0 | 2 | 0 | 0 | 0 | 1 | 0 | 2 | 0 | 3 | 12 |
| **Arias-de la Torre, 2018** | No | 1 | 2 | 2 | 1 | 0 | 1 | 1 | 0 | 1 | 0 | 2 | 0 | 3 | 14 |
| **Augner, 2018** | No | 1 | 2 | 1 | 0 | 0 | 1 | 1 | 0 | 1 | 1 | 2 | 0 | 2 | 12 |
| **Behncke, 2012** | No | 1 | 2 | 2 | 1 | 2 | 0 | 1 | 0 | 1 | 0 | 2 | 0 | 3 | 15 |
| **Belloni, 2016** | No | 1 | 2 | 2 | 0 | 2 | 0 | 1 | 0 | 1 | 0 | 2 | 0 | 3 | 14 |
| **Borson, 1986** | No | 1 | 2 | 2 | 1 | 0 | 1 | 1 | 0 | 1 | 1 | 2 | 0 | 2 | 14 |
| **Bretanha, 2015** | No | 1 | 2 | 1 | 0 | 0 | 1 | 1 | 0 | 1 | 0 | 2 | 0 | 3 | 12 |
| **Butterworth, 2006** | No | 1 | 2 | 2 | 1 | 2 | 0 | 1 | 0 | 1 | 1 | 2 | 0 | 3 | 16 |
| **Buxton, 2005** | No | 1 | 2 | 2 | 0 | 2 | 0 | 1 | 0 | 1 | 1 | 2 | 0 | 3 | 15 |
| **Calvó-Perxas, 2016** | No | 1 | 2 | 2 | 0 | 0 | 1 | 1 | 2 | 1 | 0 | 2 | 1 | 3 | 16 |
| **Calvo, 2013** | No | 1 | 2 | 2 | 1 | 2 | 0 | 1 | 2 | 1 | 0 | 2 | 1 | 3 | 18 |
| **Choi, 2013** | No | 1 | 2 | 2 | 0 | 0 | 1 | 1 | 0 | 1 | 1 | 2 | 0 | 3 | 14 |
| **Coursolle, 2010** | No | 1 | 2 | 2 | 1 | 0 | 1 | 1 | 3 | 1 | 1 | 2 | 1 | 3 | 19 |
| **Farakhan, 1984** | No | 1 | 2 | 0 | 0 | 2 | 0 | 1 | 0 | 0 | 0 | 0 | 0 | 0 | 6 |
| **Fernández-Niño, 2018** | No | 1 | 2 | 2 | 0 | 2 | 0 | 1 | 0 | 1 | 1 | 2 | 0 | 3 | 15 |
| **Fernandez, 1998** | No | 1 | 2 | 2 | 1 | 0 | 1 | 1 | 2 | 1 | 1 | 0 | 1 | 2 | 15 |
| **Gayman, 2006** | No | 1 | 2 | 2 | 1 | 2 | 0 | 1 | 2 | 1 | 1 | 2 | 0 | 3 | 18 |
| **Han, 2021** | No | 1 | 2 | 2 | 0 | 2 | 0 | 1 | 2 | 1 | 1 | 2 | 0 | 3 | 17 |
| **Heller-Sahlgren, 2017** | No | 1 | 2 | 2 | 0 | 2 | 0 | 1 | 0 | 1 | 1 | 2 | 0 | 3 | 15 |
| **Herzog, 1991** | No | 1 | 2 | 2 | 1 | 0 | 1 | 1 | 0 | 1 | 1 | 2 | 0 | 3 | 15 |
| **Kim, 2002** | No | 1 | 2 | 2 | 1 | 0 | 1 | 1 | 0 | 1 | 1 | 2 | 0 | 3 | 15 |
| **Kolodziej, 2019** | No | 1 | 2 | 2 | 0 | 2 | 0 | 1 | 0 | 1 | 1 | 2 | 0 | 3 | 15 |
| **Leinonen, 2013** | No | 1 | 2 | 1 | 0 | 2 | 0 | 1 | 3 | 1 | 1 | 2 | 1 | 3 | 18 |
| **Matta, 2020** | No | 1 | 2 | 2 | 1 | 0 | 1 | 1 | 3 | 1 | 1 | 2 | 0 | 3 | 18 |
| **Midanik 1995** | No | 1 | 2 | 2 | 0 | 2 | 0 | 1 | 0 | 1 | 0 | 2 | 0 | 3 | 14 |
| **Mojon-Azzi 2007** | No | 1 | 2 | 2 | 1 | 2 | 0 | 0 | 0 | 1 | 1 | 2 | 0 | 3 | 15 |
| **Mosca 2014** | No | 1 | 2 | 1 | 0 | 2 | 0 | 1 | 2 | 1 | 1 | 2 | 0 | 3 | 16 |
| **Noh 2019** | No | 1 | 2 | 2 | 0 | 2 | 0 | 1 | 2 | 1 | 1 | 2 | 0 | 3 | 17 |
| **Olesen 2015** | No | 1 | 2 | 2 | 1 | 2 | 0 | 0 | 3 | 1 | 1 | 2 | 1 | 3 | 19 |
| **Pahkala 1992** | No | 1 | 2 | 2 | 0 | 0 | 1 | 1 | 0 | 1 | 0 | 2 | 0 | 2 | 12 |
| **Park, 2015** | No | 1 | 2 | 2 | 1 | 2 | 0 | 1 | 2 | 1 | 1 | 2 | 1 | 3 | 19 |
| **Reitzes, 1996** | No | 1 | 2 | 1 | 1 | 2 | 0 | 1 | 2 | 1 | 0 | 2 | 1 | 2 | 16 |
| **Rhee, 2016** | No | 1 | 2 | 2 | 1 | 2 | 0 | 1 | 2 | 1 | 1 | 2 | 0 | 3 | 18 |
| **Schwingel, 2009** | No | 1 | 2 | 2 | 1 | 2 | 0 | 1 | 3 | 1 | 1 | 2 | 0 | 3 | 19 |
| **Sheppard, 2018** | No | 1 | 2 | 1 | 0 | 2 | 0 | 0 | 0 | 1 | 1 | 0 | 0 | 3 | 11 |
| **Shiba, 2017** | No | 1 | 2 | 2 | 0 | 2 | 0 | 1 | 2 | 1 | 1 | 2 | 0 | 3 | 17 |
| **Tuohy, 2005** | No | 1 | 2 | 2 | 0 | 0 | 1 | 1 | 0 | 1 | 1 | 2 | 0 | 3 | 14 |
| **van de Bogaard, 2018** | No | 1 | 2 | 2 | 1 | 2 | 0 | 1 | 2 | 1 | 1 | 2 | 1 | 3 | 19 |

Q1: Is retirement (or type of retirement) grouped together with non-retirement related categories? Yes=exclude

Q2: The primary research question/objective is clearly stated. Yes=1

Q3: Is the primary research question/objective related to the systematic review study question that…type of retirement is a risk factor for depression? Yes=2

Q4: Were study sample methods (including sample size, inclusion/exclusion criteria and power) adequately described? Yes=2

Q5: Was non-response to participation in the study addressed and/or adequately described? Yes=1

Q6: Is retirement the primary exposure or main independent variable in the study? Yes=2

Q7: Is retirement a main effect, co-variable, confounder or interaction in the study? Yes=1

Q8: Is depression a measured outcome in the study? If cause-specific is selected, enter the cause(s) in the text box. Yes=1

Q9: Did the study measure retirement (exposure) before depression (outcome)? Yes=3

Q10: Is the comparison group (or reference group) appropriate? (i.e., does it make sense?) Yes=1

Q11: Did the study make comparisons between similarly employed/retired populations? Yes=1

Q12: Were covariates/potential confounders for depression (e.g., gender, age, pre-existing health conditions) appropriately used to adjust or stratify the analysis and/or adequately described? Yes=2

Q13: Was loss to follow-up appropriately addressed and/or adequately described in the study? Yes=1

Q14: Were statistical methods appropriately used and/or adequately described to examine the retirement/depression relationship? Yes=3

**Supplementary Table 3:** Sensitivity analysis conducted excluding one dataset at a time for studies with a quality score (QS) equal to or higher than 15 and for longitudinal studies with a QS equal to or higher than 15 and using validated diagnostic tools.

| **Type of analysis** | **N. of included datasets** | **ES** | **95% CI,**  **p-value** | **N. of participants** | **Chi^2^; df** | **I^2^** | **p-value** | **Intercept*** | **t-value*** | **p-value*** |
| --- | --- | --- | --- | --- | --- | --- | --- | --- | --- | --- |
| *Overall* | **60** | **0.83** | **(0.74; 0.93),**  **0.001** | **557,111** | 895.19; 59 | **93.41** | **<0.001** | **0.53** | **0.78** | **0.439** |
| Sensitivity analysis | | | | | | | | | | |
| *QS ≥15* | **47** | **0.79** | **(0.68; 0.91),**  **0.001** | **485,092** | 808.42; 46 | **94.31** | **<0.0001** | **0.52** | **0.65** | **0.520** |
| *excluding Airagnes (a) 2016* | **46** | **0.79** | **0.86-0.92, 0.002** | **482,902** | 789.27; 45 | **94.30** | **<0.0001** | **0.52** | **0.64** | **0.523** |
| *excluding Airagnes (b) 2016* | **46** | **0.79** | **0.68-0.92, 0.003** | **478,040** | 744.30; 45 | **93.95** | **<0.0001** | **0.26** | **0.33** | **0.745** |
| *excluding Airagnes 2015* | **46** | **0.79** | **0.67-0.92, 0.003** | **475,337** | 734.87; 45 | **93.88** | **<0.0001** | **0.11** | **0.14** | **0.892** |
| *excluding Alavinia 2008* | **46** | **0.77** | **0.66-0.89, <0.0001** | **473,630** | 794.21; 45 | **94.33** | **<0.0001** | **0.42** | **0.51** | **0.611** |
| *excluding Behncke 2012* | **46** | **0.79** | **0.68-0.91, 0.001** | **483,653** | 808.35; 45 | **94.43** | **<0.0001** | **0.53** | **0.64** | **0.525** |
| *excluding Butterworth (a) 2006* | **46** | **0.81** | **0.70-0.93, 0.004** | **485,092** | 790.27; 45 | **94.32** | **<0.0001** | **0.65** | **0.81** | **0.424** |
| *excluding Butterwortj (b) 2006* | **46** | **0.80** | **0.69-0.92, 0.002** | **485,092** | 805.86; 45 | **94.42** | **<0.0001** | **0.59** | **0.71** | **0.480** |
| *excluding Buxton (a) 2005* | **46** | **0.78** | **0.67-0.89, <0.0001** | **484,132** | 803.16; 45 | **94.40** | **<0.0001** | **0.46** | **0.56** | **0.579** |
| *excluding Buxton (b) 2005* | **46** | **0.77** | **0.67-0.89, <0.0001** | **484,177** | 795.14; 45 | **94.34** | **<0.0001** | **0.42** | **0.51** | **0.614** |
| *excluding Calvo 2013* | **46** | **0.82** | **0.71-0.94, 0.005** | **478,468** | 771.24; 45 | **94.17** | **<0.0001** | **0.70** | **0.87** | **0.388** |
| *excluding Calvó-Perxas (a) 2016* | **46** | **0.78** | **0.67-0.90, 0.001** | **476,599** | 800.83; 45 | **94.38** | **<0.0001** | **0.48** | **0.59** | **0.560** |
| *excluding Calvó-Perxas (b) 2016* | **46** | **0.78** | **0.67-0.90, 0.001** | **477,666** | 805.21; 45 | **94.41** | **<0.0001** | **0.49** | **0.59** | **0.556** |
| *excluding Coursolle (a) 2010* | **46** | **0.80** | **0.69-0.92, 0.002** | **483,892** | 803.36; 45 | **94.40** | **<0.0001** | **0.60** | **0.74** | **0.466** |
| *excluding Coursolle (b)2010* | **46** | **0.80** | **0.70-0.93** | **483,626** | 789.60; 45 | **94.37** | **<0.0001** | **0.64** | **0.78** | **0.441** |
| *excluding Fernández-Niño (a) 2018* | **46** | **0.78** | **0.67-0.90, 0.001** | **481,238** | 804.97; 45 | **94.41** | **<0.0001** | **0.48** | **0.58** | **0.566** |
| *excluding Fernández-Niño (b) 2018* | **46** | **0.80** | **0.69-0.92, 0.002** | **481,421** | 803.78; 45 | **94.40** | **<0.0001** | **0.61** | **0.74** | **0.462** |
| *excluding Fernández-Niño (c) 2018* | **46** | **0.78** | **0.67-0.90, 0.001** | **483,623** | 803.96; 45 | **94.40** | **<0.0001** | **0.47** | **0.56** | **0.575** |
| *excluding Fernández-Niño (d) 2018* | **46** | **0.79** | **0.69-0.92, 0.002** | **483,718** | 805.64; 45 | **94.41** | **<0.0001** | **0.60** | **0.72** | **0.474** |
| *excluding Fernández-Niño (e) 2018* | **46** | **0.80** | **0.69-0.92, 0.002** | **483,222** | 801.08; 45 | **94.38** | **<0.0001** | **0.64** | **0.77** | **0.443** |
| *excluding Fernández-Niño (f) 2018* | **46** | **0.79** | **0.68-0.91, 0.001** | **482,991** | 807.85; 45 | **94.43** | **<0.0001** | **0.56** | **0.68** | **0.498** |
| *excluding Fernández-Niño (g) 2018* | **46** | **0.79** | **0.68-0.91, 0.001** | **483,947** | 808.08; 45 | **94.43** | **<0.0001** | **0.56** | **0.68** | **0.501** |
| *excluding Fernández-Niño (h) 2018* | **46** | **0.78** | **0.68-0.91, 0.001** | **483,169** | 808.28; 45 | **94.43** | **<0.0001** | **0.52** | **0.64** | **0.529** |
| *excluding Fernández-Niño (i) 2018* | **46** | **0.78** | **0.68-0.90, 0.001** | **483,970** | 807.87; 45 | **94.43** | **<0.0001** | **0.51** | **0.62** | **0.538** |
| *excluding Fernández-Niño (j) 2018* | **46** | **0.79** | **0.69-0.92, 0.002** | **483,828** | 805.53; 45 | **94.41** | **<0.0001** | **0.60** | **0.73** | **0.472** |
| *excluding Fernández-Niño (k) 2018* | **46** | **0.79** | **0.68-0.91, 0.001** | **484,211** | 808.41; 45 | **94.43** | **<0.0001** | **0.53** | **0.65** | **0.521** |
| *excluding Gayman (a) 2013* | **46** | **0.78** | **0.67-0.90, 0.001** | **482,327** | 766.60; 45 | **94.13** | **<0.0001** | **0.48** | **0.61** | **0.547** |
| *excluding Gayman (b) 2013* | **46** | **0.78** | **0.67-0.90, 0.001** | **484,593** | 805.16; 45 | **94.41** | **<0.0001** | **0.49** | **0.59** | **0.555** |
| *excluding Han 2021* | **46** | **0.79** | **0.68-0.91, 0.002** | **475,745** | 807.58; 45 | **94.43** | **<0.0001** | **0.54** | **0.67** | **0.508** |
| *excluding Heller-Sahlgren 2017* | **46** | **0.77** | **0.67-0.89, <0.0001** | **480,388** | 789.44; 45 | **94.30** | **<0.0001** | **0.43** | **0.53** | **0.599** |
| *excluding Kim (a) 2002* | **46** | **0.79** | **0.68-0.91, 0.001** | **484,916** | 807.92; 45; 45 | **94.43** | **<0.0001** | **0.57** | **0.69** | **0.497** |
| *excluding Kim (b) 2002* | **46** | **0.79** | **0.68-0.91, 0.001** | **484,810** | 807.92; 45 | **94.43** | **<0.0001** | **0.57** | **0.68** | **0.497** |
| *excluding Kolodziej (a) 2019* | **46** | **0.80** | **0.70-0.93, 0.003** | **467,807** | 798.90; 45 | **94.37** | **<0.0001** | **0.63** | **0.77** | **0.444** |
| *excluding Kolodziej (b) 2019* | **46** | **0.79** | **0.69-0.92, 0.002** | **465, 082** | 806.51; 45 | **94.42** | **<0.0001** | **0.58** | **0.71** | **0.483** |
| *excluding Matta 2020* | **46** | **0.83** | **0.73-0.95, 0.008** | **467,437** | 678.19; 45 | **93.36** | **<0.0001** | **0.76** | **1.02** | **0.313** |
| *excluding Mojon-Azzi 2007* | **46** | **0.78** | **0.67-0.90, 0.001** | **484,535** | 792.36; 45 | **94.32** | **<0.0001** | **0.47** | **0.58** | **0.566** |
| *excluding Mosca 2014* | **46** | **0.78** | **0.68-0.91, 0.001** | **482,719** | 808.06; 45 | **94.43** | **<0.0001** | **0.52** | **0.63** | **0.535** |
| *excluding Noh 2019* | **46** | **0.78** | **0.67-0.90, 0.001** | **477,958** | 803.92; 45 | **94.40** | **<0.0001** | **0.48** | **0.58** | **0.564** |
| *excluding Olesen (a) 2015* | **46** | **0.77** | **0.67-0.90, 0.001** | **363,878** | 790.57; 45 | **94.31** | **<0.0001** | **0.45** | **0.55** | **0.583** |
| *excluding Olesen (b) 2015* | **46** | **0.78** | **0.67-0.90, 0.001** | **361,224** | 799.01; 45 | **94.37** | **<0.0001** | **0.47** | **0.58** | **0.566** |
| *excluding Park (a) 2016* | **46** | **0.77** | **0.67-0.89, <0.0001** | **484,390** | 778.42; 45 | **94.22** | **<0.0001** | **0.41** | **0.51** | **0.611** |
| *excluding Park (b) 2016* | **46** | **0.77** | **0.67-0.89, <0.0001** | **483,462** | 781.57; 45 | **94.24** | **<0.0001** | **0.44** | **0.54** | **0.589** |
| *excluding Reitzes 1996* | **46** | **0.79** | **0.68-0.91, 0.001** | **484,335** | 808.42; 45 | **94.43** | **<0.0001** | **0.54** | **0.65** | **0.520** |
| *excluding Rhee 2016* | **46** | **0.78** | **0.67-0.90, 0.001** | **483,897** | 782.15; 45 | **94.25** | **<0.0001** | **0.48** | **0.60** | **0.549** |
| *excluding Schwingel 2009* | **46** | **0.79** | **0.69-0.92, 0.001** | **483,338** | 774.32; 45 | **94.19** | **<0.0001** | **0.49** | **0.61** | **0.544** |
| *excluding Shiba (a) 2017* | **46** | **0.77** | **0.67-0.89, <0.0001** | **451,523** | 759.82; 45 | **94.08** | **<0.0001** | **0.45** | **0.57** | **0.573** |
| *excluding Shiba (b) 2017* | **46** | **0.77** | **0.67-0.89, <0.0001** | **456,224** | 709.26; 45 | **93.66** | **<0.0001** | **0.49** | **0.65** | **0.522** |
| *excluding van den Bogaard 2018* | **46** | **0.78** | **0.67-0.91, 0.002** | **476,000** | 771.36; 45 | **94.17** | **<0.0001** | **0.75** | **0.94** | **0.355** |
| *QS ≥15 + validated tool to diagnose depression + longitudinal design* | **24** | **0.76** | **(0.64; 0.90), 0.001** | **162,004** | 652.18; 23 | **96.47** | **<0.001** | **0.85** | **0.52** | **0.607** |
| *excluding Airagnes (a) 20016* | **23** | **0.77** | **(0.64-0.92), 0.004** | **159,814** | 636.31; 22 | **96.54** | **<0.0001** | **0.90** | **0.55** | **0.590** |
| *excluding Airagnes (b) 2016* | **23** | **0.77** | **(0.63-0.92), 0.005** | **154,952** | 598.97; 22 | **96.33** | **<0.0001** | **0.46** | **0.28** | **0.784** |
| *excluding Airagnes (2015)* | **23** | **0.76** | **(0.63-0.92), 0.006** | **152,249** | 592.60; 22 | **96.29** | **<0.0001** | **0.18** | **0.11** | **0.915** |
| *excluding Calvo 2013* | **23** | **0.80** | **(0.68-0.95), 0.012** | **155,380** | 616.23; 22 | **96.43** | **<0.0001** | **1.35** | **0.81** | **0.425** |
| *excluding Calvó-Perxas (a) 2016* | **23** | **0.75** | **(0.63-0.89), 0.001** | **153,511** | 643.42; 22 | **96.58** | **<0.0001** | **0.72** | **0.43** | **0.673** |
| *excluding Calvó-Perxas (b) 2016* | **23** | **0.75** | **(0.63-089), 0.001** | **154,578** | 648.49; 22 | **96.61** | **<0.0001** | **0.76** | **0.45** | **0.660** |
| *excluding Coursolle (a) 2010* | **23** | **0.78** | **(0.65-0.92), 0.004** | **160,804** | 647.43; 22 | **96.60** | **<0.0001** | **1.09** | **0.64** | **0.532** |
| *excluding Coursolle (b) 2010* | **23** | **0.78** | **(0.66-0.93), 0.006** | **160,538** | 642.69; 22 | **96.58** | **<0.0001** | **1.19** | **0.69** | **0.497** |
| *excluding Gayman (a) 2013* | **23** | **0.74** | **(0.62-0.88), 0.001** | **159,239** | 605.98; 22 | **96.37** | **<0.0001** | **0.67** | **0.41** | **0.684** |
| *excluding Gayman (b) 2013* | **23** | **0.75** | **(0.63-0.89), 0.001** | **161,505** | 648.41; 22 | **96.61** | **<0.0001** | **0.76** | **0.45** | **0.660** |
| *excluding Han 2021* | **23** | **0.76** | **(0.64-0.91), 0.003** | **152,657** | 651.63; 22 | **96.62** | **<0.0001** | **0.92** | **0.54** | **0.593** |
| *excluding Kim (a) 2002* | **23** | **0.76** | **(0.64-0.90), 0.002** | **161,828** | 651.69; 22 | **96.62** | **<0.0001** | **1.01** | **0.57** | **0.572** |
| *excluding Kim (b) 2002* | **23** | **0.76** | **(0.64-0.90), 0.002** | **161,722** | 651.68; 22 | **96.62** | **<0.0001** | **1.00** | **0.57** | **0.572** |
| *excluding Matta 2020* | **23** | **0.82** | **(0.70-0.97), 0.018** | **144,349** | 525.75; 22 | **95.82** | **<0.001** | **1.55** | **1.03** | **0.315** |
| *excluding Mosca 2014* | **23** | **0.76** | **(0.64-0.90), 0.001** | **159,631** | 651.79; 22 | **96.62** | **<0.0001** | **0.87** | **0.50** | **0.623** |
| *excluding Noh 2019* | **23** | **0.75** | **(0.63-0.89), 0.001** | **154,870** | 647.22; 22 | **96.60** | **<0.0001** | **0.74** | **0.43** | **0.671** |
| *excluding Park (a) 2016* | **23** | **0.73** | **(0.62-0.87), <0.0001** | **161,302** | 620.35; 22 | **96.45** | **<0.0001** | **0.54** | **0.32** | **0.749** |
| *excluding Park (b) 2016* | **23** | **0.74** | **(0.62-0.88), 0.001** | **160,374** | 623.12; 22 | **96.47** | **<0.0001** | **0.60** | **0.36** | **0.722** |
| *excluding Reitzes 1996* | **23** | **0.76** | **(0.64-0.90), 0.002** | **161,247** | 652.17; 22 | **96.63** | **<0.0001** | **0.93** | **0.53** | **0.603** |
| *excluding Rhee 2016* | **23** | **0.74** | **(0.62-0.89), 0.001** | **160,809** | 622.59; 22 | **96.47** | **<0.0001** | **0.70** | **0.42** | **0.677** |
| *excluding Schwingel* | **23** | **0.77** | **(0.64-0.92), 0.004** | **160,250** | 623.04; 22 | **96.47** | **<0.0001** | **0.86** | **0.53** | **0.604** |
| *excluding Shiba (a) 2017* | **23** | **0.74** | **(0.62-0.87), <0.0001** | **128,435** | 599.59; 22 | **96.33** | **<0.0001** | **0.60** | **0.37** | **0.718** |
| *excluding Shiba (b) 2017* | **23** | **0.74** | **(0.62-0.87), <0.0001** | **133,136** | 545.43; 22 | **95.97** | **<0.0001** | **0.64** | **0.42** | **0.680** |
| *excluding van den Bogaard 2018* | **23** | **0.75** | **(0.62-0.90), 0.002** | **152,912** | 604.41; 22 | **96.36** | **<0.0001** | **1.30** | **0.80** | **0.435** |
| * Egger’s linear regression test; df= degree of freedom; ES= Effect Size; N.= number; QS= quality score | | | | | | | | | | |

**Supplementary Figure 1:** Forest plot of sensitivity analysis for the meta-analysis assessing the association between retirement and depression: **a)** studies with a quality score (QS) equal to or higher than 15 and **b)** studies with a quality score (QS) equal to or higher than 15 and using validated diagnostic tools. ES, effect size; CI, confidence interval.


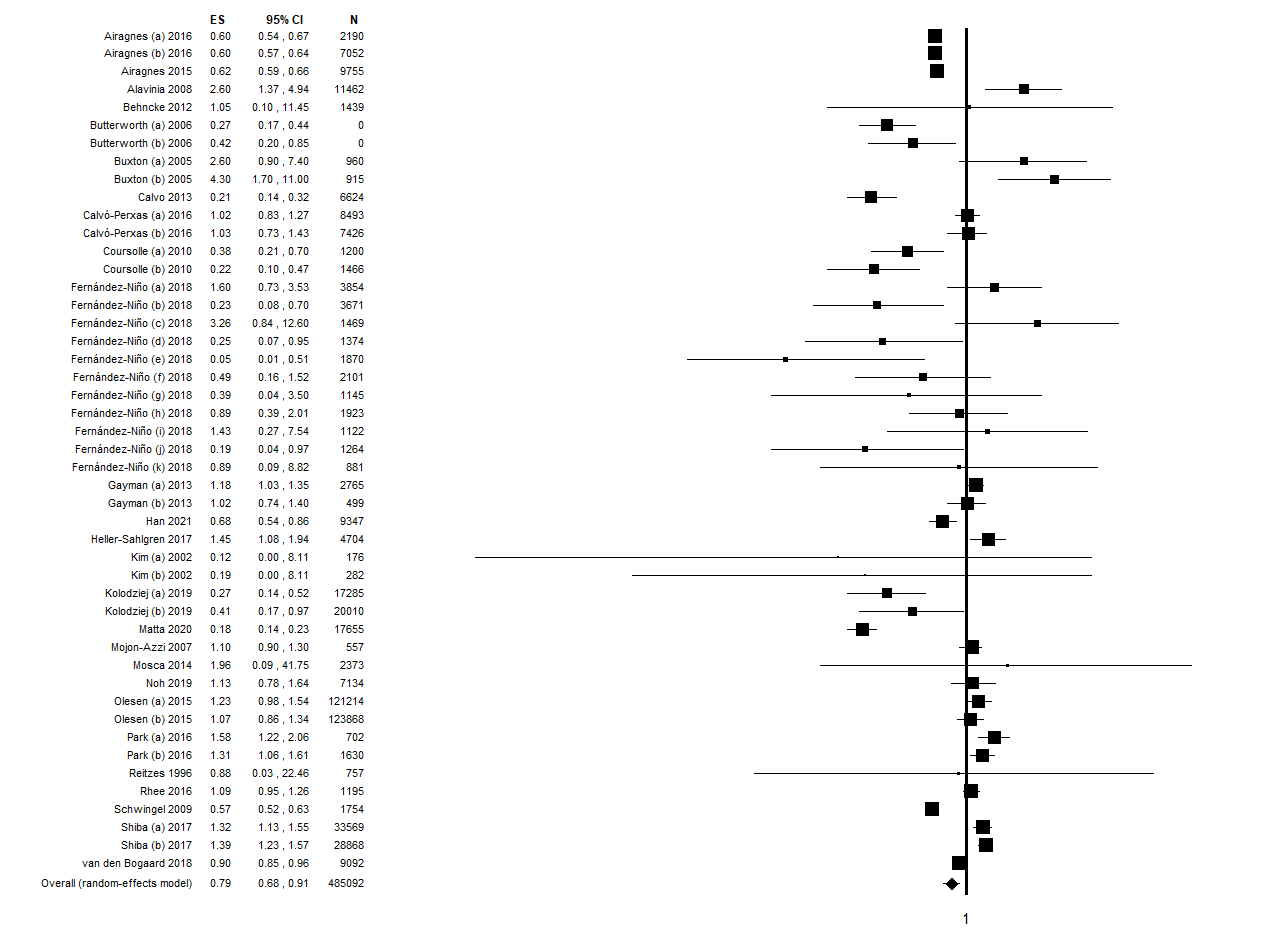


1. QS≥15


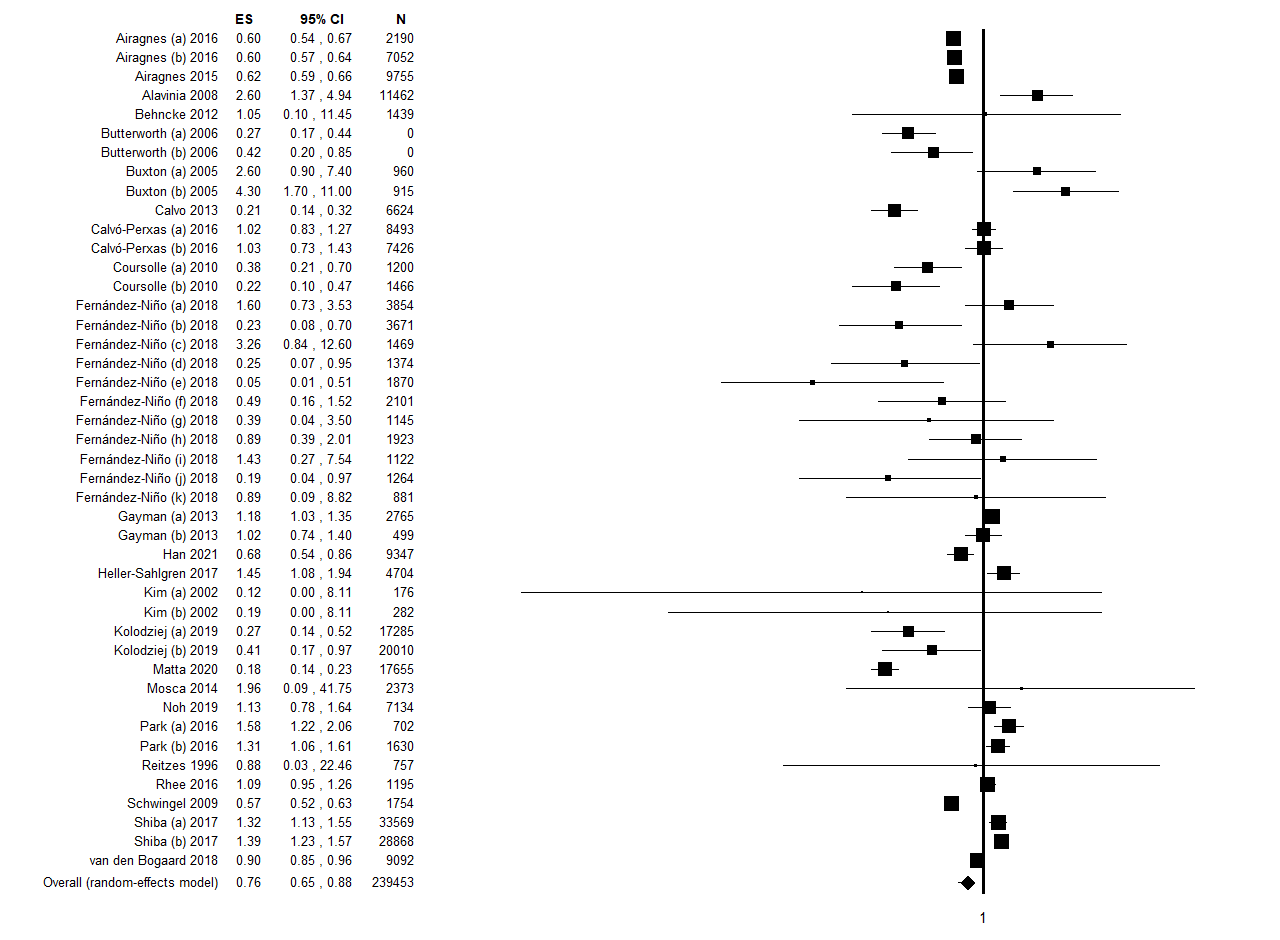


1. QS≥15 + validated diagnostic tool

**Supplementary Figure 2:** Forest plot of subgroups analysis for the meta-analysis assessing the association between retirement and depression: **a)** cross-sectional studies, **b)** among women and **c)** among men. ES, effect size; CI, confidence interval.


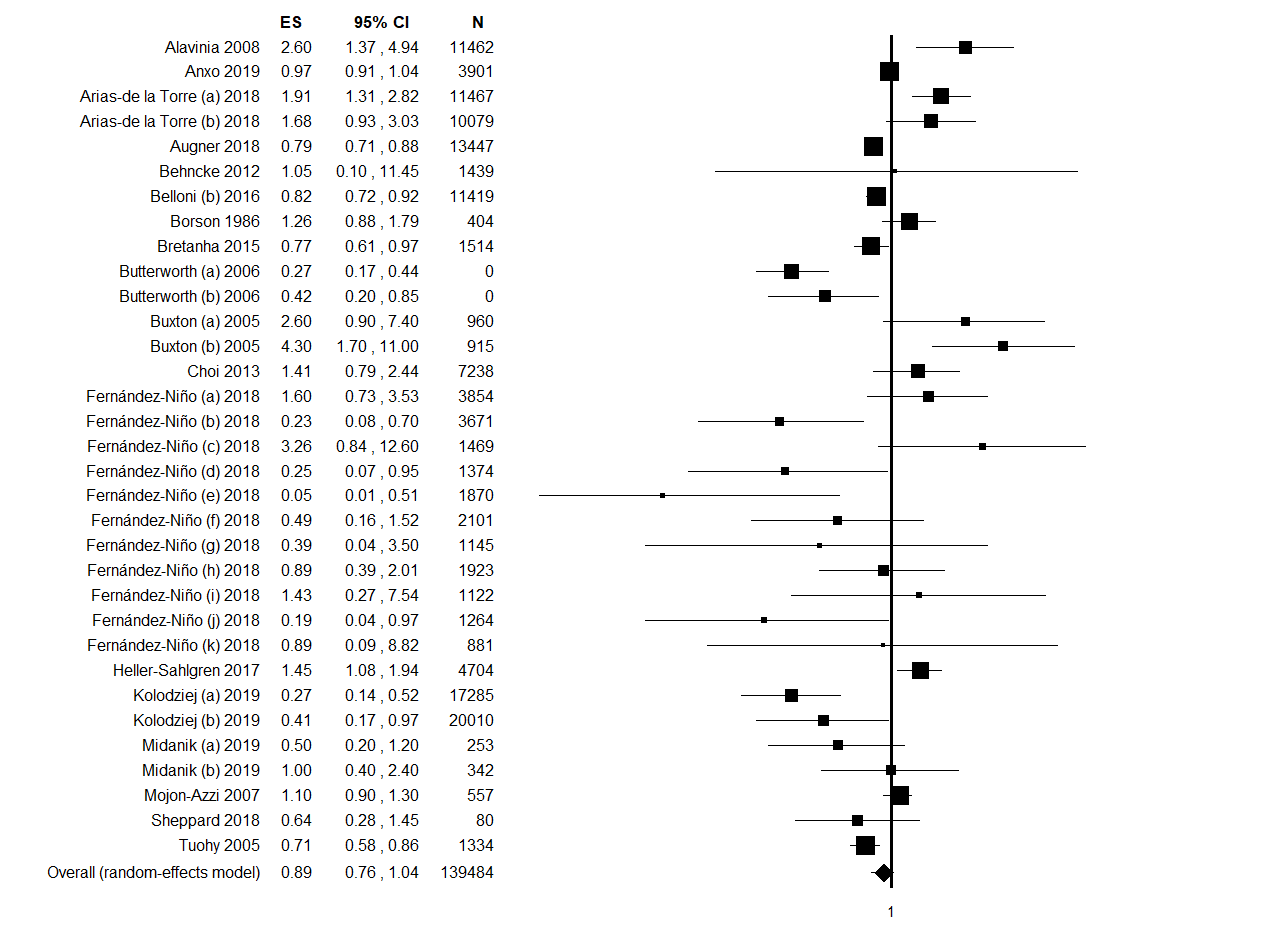


1. cross-sectional studies


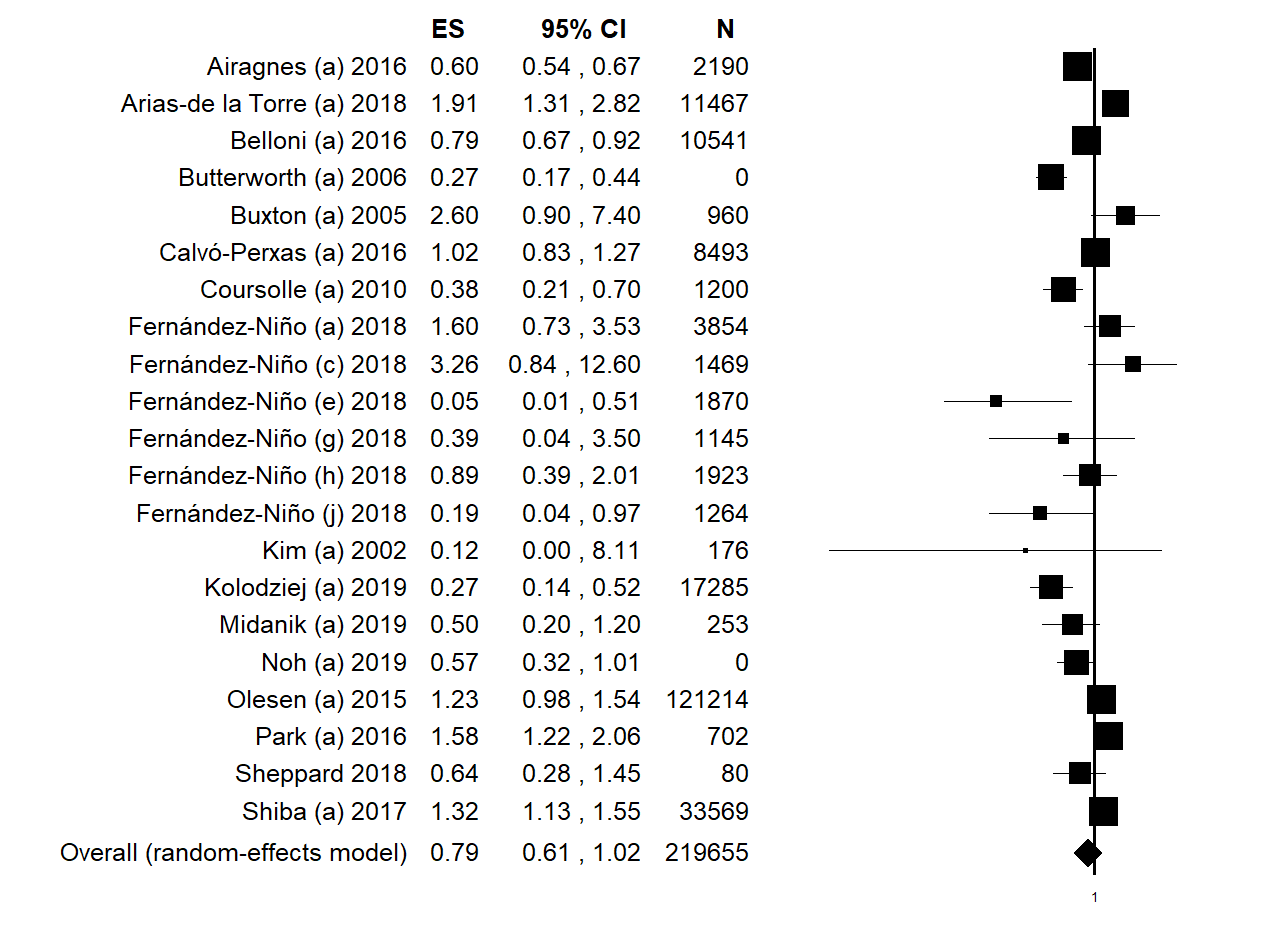


| **b)** women |
| --- |
|  |


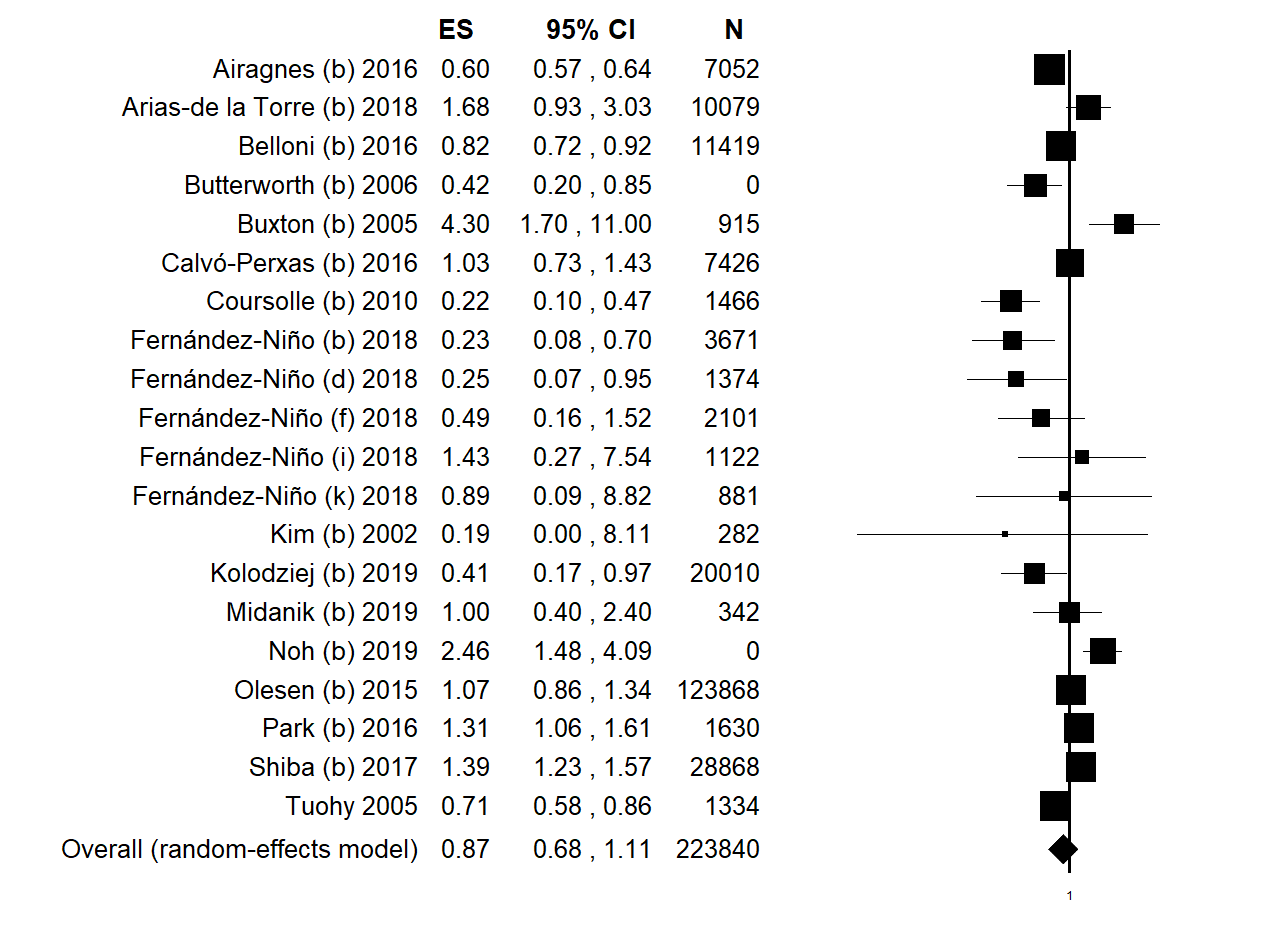


**c)** men
